# Biodegradable Polymeric Nanoparticles for Drug Delivery to Solid Tumors

**DOI:** 10.3389/fphar.2021.601626

**Published:** 2021-02-03

**Authors:** Agnese Gagliardi, Elena Giuliano, Eeda Venkateswararao, Massimo Fresta, Stefania Bulotta, Vibhudutta Awasthi, Donato Cosco

**Affiliations:** ^1^ Department of Health Sciences, University “Magna Græcia” of Catanzaro, Catanzaro, Italy; ^2^ Department of Pharmaceutical Sciences, The University of Oklahoma Health Sciences Center, Oklahoma City, OK, United States

**Keywords:** surfactants, PEG, polymeric nanoparticles, passive targeting, cancer

## Abstract

Advances in nanotechnology have favored the development of novel colloidal formulations able to modulate the pharmacological and biopharmaceutical properties of drugs. The peculiar physico-chemical and technological properties of nanomaterial-based therapeutics have allowed for several successful applications in the treatment of cancer. The size, shape, charge and patterning of nanoscale therapeutic molecules are parameters that need to be investigated and modulated in order to promote and optimize cell and tissue interaction. In this review, the use of polymeric nanoparticles as drug delivery systems of anticancer compounds, their physico-chemical properties and their ability to be efficiently localized in specific tumor tissues have been described. The nanoencapsulation of antitumor active compounds in polymeric systems is a promising approach to improve the efficacy of various tumor treatments.

## Introduction

Cancer is the second leading cause of death in the world, and was responsible for approximately 9.6 million deaths in 2018 (Bray et al., 2018). Over the next 20 years, the number of new cases is estimated to increase by about 70% (Siegel et al., 2017). Cancer therapy is considered a multidisciplinary challenge requiring close collaboration among clinicians, biologists, and biomedical engineers (Danhier et al., 2010). Current cancer treatments include surgery, radiation, and chemotherapy, but the effects of these procedures may cause damage to normal as well as tumoral cells. The resultant systemic toxicity and adverse effects greatly limit the maximum tolerated dose of anti-cancer drugs, and thus restrict their therapeutic efficacy. In particular, surgery together with radiotherapy are the first choice used for local and non-metastatic cancers, while anti-cancer drugs (chemotherapy, hormone, and biological therapies) are the treatments currently employed in metastatic cancers and adjuvant therapies (Tran et al., 2017). The toxicity of conventional chemotherapeutic drugs, as well as the indiscriminate destruction of healthy cells and the development of multidrug resistance, are the motivating thrust behind research on novel targeted treatments (Pérez-Herrero and Fernández-Medarde, 2015; Tran et al., 2017). The main challenge is to improve the selectivity of anticancer drugs for tumor cells and the tumor microenvironment, while sparing healthy cells and tissues. In this context, a promising approach is the targeting of tumor tissue by nanomedicine-based therapeutics (Oerlemans et al., 2010). These formulations are made up of submicrometer-sized carriers containing the active compound(s), which are able to selectively diagnose and treat tumors by suitable targeting vectors, thus improving the therapeutic index and the pharmacokinetic profile of the anticancer drugs that are delivered.

Nanocarriers can retain multiple therapeutic agents not only to enhance their therapeutic effect on a synergestic or additive basis, but also to overcome acquired resistance to single chemotherapeutic drugs. Many tumors develop chemo-resistance through many mechanisms, including induction of the drug efflux rate or the downregulation of uptake mechanisms (Mansoori et al., 2017). Nanoparticulate formulations can overcome this limitation by providing an alternative pathway of cellular internalization. Currently, several therapeutic nanoparticle platforms are being investigated for targeted cancer treatment, including lipid-based, polymer-based, inorganic, viral, and polymer-drug conjugated systems. In the past two decades, over 20 nanotechnology-based therapeutic products have been approved for clinical use. Among these products, liposomal systems and polymer-drug conjugates are two of the most important groups, and many other formulations are under clinical investigation, including chemotherapy, hyperthermia, radiation therapy, gene or RNA interference (RNAi) therapy, and immunotherapy (Wicki et al., 2015).

Nanocarriers have unique features such as their nanometric size, high surface area-to-volume ratio, favorable drug release profiles and targeting features which can promote their preferential accumulation in tumor tissues (Wicki et al., 2015). Most nanosystems for the treatment of solid tumors are administered systemically and accumulated in the tumor tissues through the enhanced permeability and retention (EPR) effect, which is generally thought to be the result of leaky tumor vasculature and poor lymphatic drainage (Maeda, 2015). However, this interpretation of EPR-dependence is simplistic, because the biodistribution of systemically administerd nanosystems can be influenced by multiple biological factors, including interaction with plasma proteins, blood circulation time, extravasation, penetration of tumor tissue, and cancer cell uptake (Shi et al., 2017). Modification of the surfaces of the nano-systems—which are able to confer specific targeting properties or stimuli-sensitive responses—also affect their overall distribution.

**FIGURE 1:**
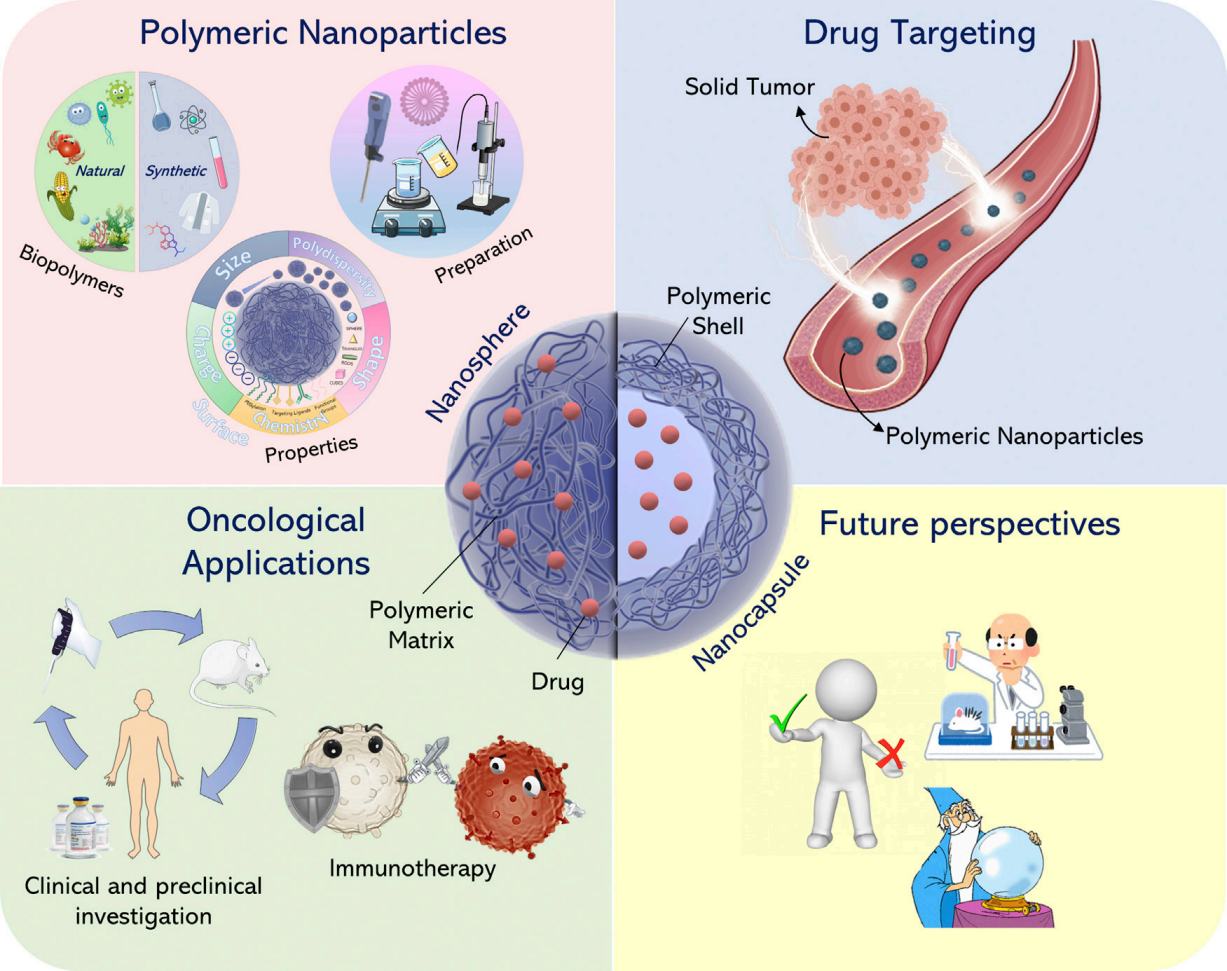
Overview of the main features of polymeric nanoparticles.

Much of our current knowledge regarding the *in vivo* behavior of nanoparticulate systems is based on data obtained from animal models. But relatively few investigations have correlated the obtained data in order to determine whether and how the safety and the efficacy of nanoparticles in humans can be better predicted by using these animal models (Hrkach et al., 2012; Zuckerman et al., 2014). There also exist a number of scientific articles which focus on specific aspects and applications concerning the development of polymeric nanoparticles. Therefore, this work is not intended to be a review of all the research performed in this area, but rather to provide the basic concepts and ideas related to the preparation and use of polymer-based nanoparticles as drug carriers in cancer therapy. This article offers an overview and discusses the most important findings and prospects as illustrated in Figure 1.

## Tumor Microenvironment

**FIGURE 2:**
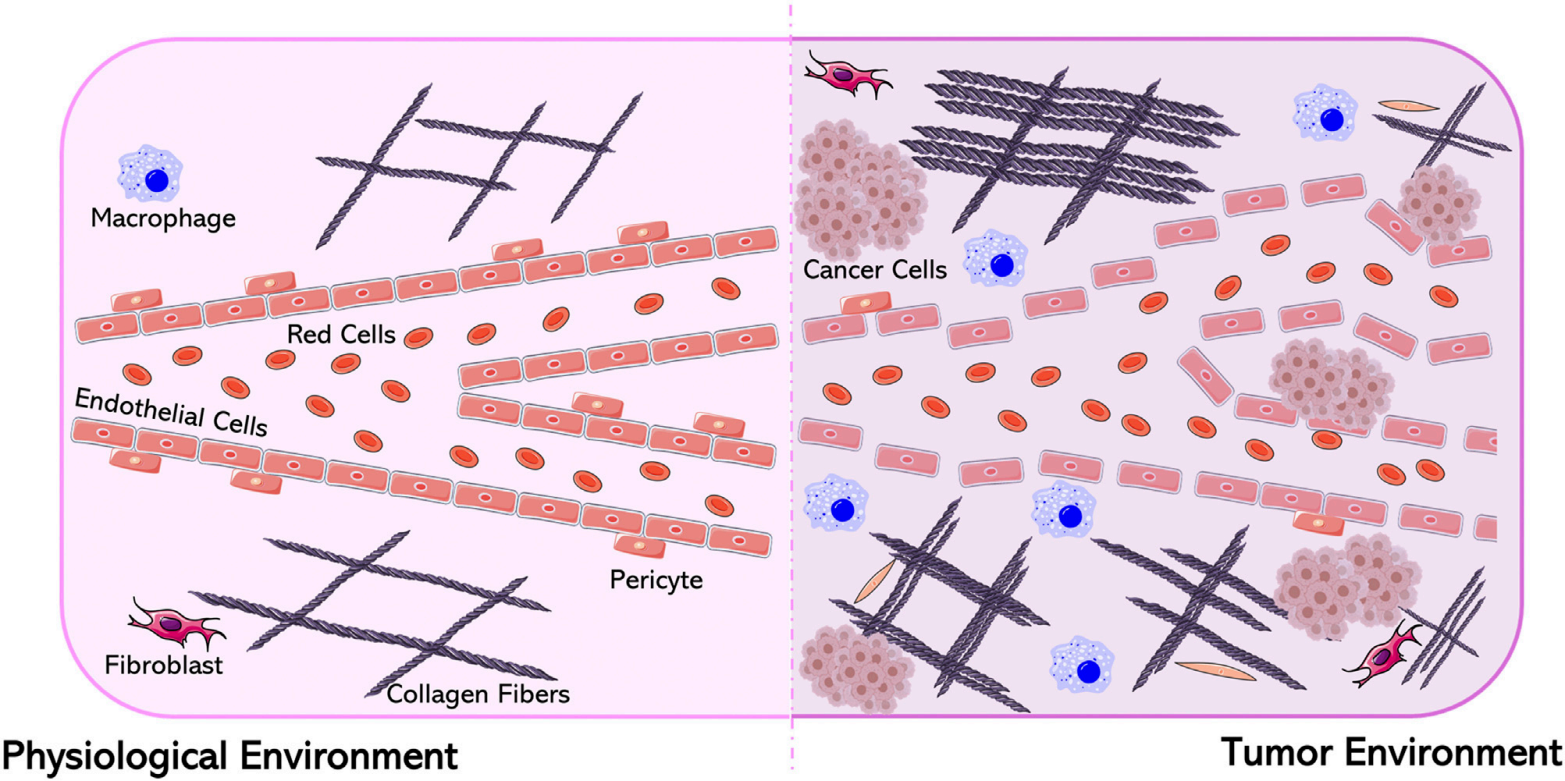
Differences between a physiological and a tumor environment. Figure generated from Servier Medical Art.

The microenvironment of tumor tissue significantly differs from that of healthy tissue. These differences include vascular abnormalities, oxygenation and perfusion levels, pH, and metabolic status (Abadjian et al., 2017). Solid tumors are characterized by a heterogeneous population of neoplastic cells supplied by an irregular and discontinuous endothelium with large gaps between the endothelial cells, and abnormally thick or thin basement membranes where pericites are loosely attached to endothelial cells (Figure 2). The irregularity of tumor blood vessels in their distribution, diameter, density, and serpentine shape, can be the cause of poor perfusion which leads to excessive fluid extravasation (Khawar et al., 2015). The two main causes of this heterogeneity are spatial stress, resulting from rapid tumor growth, and the abnormal extracellular matrix which can compress the vessels and partially block the flow of blood [which causes the escape of plasma and a high interstitial fluid pressure (IFP)] (Jain, 2013). The IFP is highest at the center of solid tumors and decreases radially, creating a movement of fluid away from the central region of the tumor. This phenomenon contributes to a reduced transcapillary transport of therapeutic drugs as well as their scarce accumulation in the middle of the tumor (Danhier et al., 2010). The elevated IFP and associated peritumoral edema also assist in the transport of growth factors and cancer cells away from the tumor, thus favoring tumor progression, while the abnormal and disorganized tumor vasculature results in inefficient blood flow inside the tumor mass, hypoxia, and low extracellular pH (Khawar et al., 2015).

Hypoxia plays a crucial role in tumor growth and metastasis through the induction of molecular signaling which is responsible for genetic instability, inflammation, immunosuppression, epithelial–mesenchymal transition and altered metabolism (Jing et al., 2019). It also confers resistance against several kinds of treatment, such as radiation, chemo-, photodynamic and immunotherapies, which require oxygen for efficacy (Jain, 2013). By virtue of the hypoxia-inducible factor-mediated pathway, hypoxia promotes angiogenesis. Oxygen can diffuse for maximum 150 µm beyond the capillary wall, which implies that when a tumor reaches a certain size (∼2 mm^3^), a state of cellular hypoxia begins. Angiogenesis is a cellular mechanism which is upregulated in tumoral microenvironments and creates new blood vessels to further assist tumor growth by supplying oxygen and nutrients (Jászai and Schmidt, 2019). This process consists of five steps: i) endothelial cell activation, ii) basement membrane degradation, iii) endothelial cell migration, iv) new vessel formation, and v) angiogenic remodeling. In the first phase, hypoxia induces an increase of the hypoxia-inducible factor-mediated transcription of pro-angiogenic proteins such as vascular endothelial growth factor (VEGF), platelet-derived growth factor (PDGF), and tumor necrosis factor-α (TNF-α) (Wigerup et al., 2016). Activated endothelium regulates the migration of endothelial cells through the extracellular matrix during vessel formation, due to the expression of a dimeric transmembrane integrin αvβ3 which interacts with the proteins of the extracellular matrix (vitronectin, fibronectin, etc.) (Demircioglu and Hodivala-Dilke, 2016). Successively, the matrix metalloproteinases synthesized by the activated endothelial cells degrade the basement membrane and the extracellular matrix. This process causes the apoptosis of the inner layer of endothelial cells, leading to the formation of a vessel lumen and remodeling of the immature vasculature, stabilized by pericytes and smooth-muscle cells. Often this step remains incomplete, resulting in irregularly-shaped, dilated, and tortuous tumor blood vessels. The angiogenic switch is the crucial phase in which a tumor changes from a non-angiogenic to an angiogenic phenotype and allows the dissemination of cancer cells throughout the body (Jászai and Schmidt, 2019).

Hypoxia also results in metabolic acidosis caused by increased glycolysis (the Warburg effect); this lowers the extracellular pH to 6.0–7.0 (De Palma et al., 2017). Acidosis is also a factor in epithelial–mesenchymal transition and it synergistically contributes to tumor invasion and metastasis (Yang et al., 2016). Because of their rapid growth, tumor cells continue to exploit glycolysis as an ATP-generating pathway even when oxygen is available, lowering dependence on glucose oxidation for energy production (Fu et al., 2017). This metabolic preference is mostly due to defective mitochondrial function (Kim et al., 2009). The elevated breakdown of glucose produces large amounts of lactic acid and significant amounts of free protons (H+) which are pumped into the extracellular milieu by mechanisms involving the carbonic anhydrases IX and XII (Martinez-Outschoorn et al., 2017). The resulting pH gradients between intra- and extracellular compartments within the tumor tissue, as well as between the tumor mass and the general host tissue, are potential sources of variable and often inefficient partitioning and distribution of drugs. Exposure to chemotherapy may favor the selection of tumor-cell clones with acidic organelles, which are able to entrap the drugs, and if these organelles are part of the secretory pathway, then the drug will be transported out of the cell through exocytosis. All these factors in the tumor microenvironment contribute to multidrug resistance (MDR) phenomena (Danhier et al., 2010).

## Polymeric Nanoparticles

Over the last decade nanoparticles have become extremely attractive for application in biology and medicine (Mogoşanu et al., 2016). They have the potential to modulate biopharmaceutical features, pharmacokinetic properties, and the therapeutic efficacy of entrapped drugs (Dang and Guan, 2020). Technically, nanoparticles are defined as being less than 100 nm, but in practice structures up to 300 nm in size are included in this category (Guo et al., 2016), and they can fall into different classes as a function of their morphology, size, composition, and physicochemical properties (Khan et al., 2019).

Polymer-based nanoparticles are colloidal systems made up of natural or synthetic polymers. They furnish significant advantages over other nanocarriers such as liposomes, micelles and inorganic nanosystems, and include the feasibility of scale-up and the manufacturing process under Good Manufacturing Practices (GMP) (van Vlerken et al., 2007). Other peculiar characteristics of polymeric nanoparticles are the significant stability of polymeric nanoparticles in biological fluids along with the wide availability of various polymers, the opportunity to functionalize their surfaces and to modulate polymer degradation and the leakge of the entrapped compound(s) as a function of specific stimuli (Venkatraman et al., 2010; Goodall et al., 2015; Sarcan et al., 2018).

Several chemotherapeutics have been encapsulated in polymeric delivery systems, with the aim of increasing antitumor efficacy, inhibiting metastases, and decreasing the effective dose and side effects. Polymers can encapsulate an active compound within their structure or adsorb it onto their surfaces (Masood, 2016). Langer and Folkman were the first to demonstrate the controlled release of macromolecules using polymers, which allowed the development of antiangiogenic drug delivery systems for cancer therapy (Langer and Folkman, 1976).

Ideally, the polymers selected for parenteral administration must be biocompatible, biodegradable, and possess specific mechanical and physicochemical properties (Vilar et al., 2012). The first polymers used to develop polymeric nanoparticles (PNs) were non-biodegradable polymers, such as poly(methyl methacrylate) (PMMA), polyacrylamide, polystyrene, and polyacrylates. The nanosystems made up of these materials exhibited a rapid and efficient clearance, but chronic toxicity and inflammatory reactions were observed.

Usually, non-degradable polymers require degradation times longer than their effective duration of application (Anju et al., 2020), whereas the degradation rate of biodegradable polymeric nanoparticles can be influenced by several parameters, including their physico-chemical properties (size, structure, molecular weight) and external factors, such as pH and temperature (Su and Kang, 2020). Although pioneering studies on polymeric nanoparticles have focused on non-degradable materials, the use of biodegradable polymers had a great impact as a consequence of their notable biocompatibility and biosafety (Kamaly et al., 2016).

Biodegradable polymers include synthetic polymers such as poly(D,l-lactide) (PLA), poly(D,L-glycolide) (PLG), co-polymer poly(lactide-co-glycolide) (PLGA), polyalkylcyanoacrylates, poly-Ɛ-caprolactone. They are considered safe and a few biodegradable polymer products have been approved by the US Food and Drug Administration (FDA) as well as by the European Medicines Agency (EMA) for pharmaceutical application (Palma et al., 2018). In general, biodegradable polymeric particles show reduced systemic toxicity, are more biocompatible, and favor modulation of drug-release kinetics. They are typically degraded into oligomers and monomers, which are further metabolized and eliminated from the body via normal pathways (Ravivarapu et al., 2006; Vilar et al., 2012). Non-synthetic biodegradable polymers, which include natural polymers such as chitosan, alginate, gelatin, zein, and albumin, have also been used to prepare polymeric nanoparticles (Gagliardi et al., 2018). We will discuss commonly-used polymers for the preparation of drug-loaded PNs for anticancer therapy later.

### Biopolymers for Cancer Nanomedicine

Biopolymers are one of the most important classes of biomaterials (Anju et al., 2020) and are widely used in biomedical applications because of their biocompatibility and biodegradability (Jaimes-Aguirre et al., 2016). They are macromolecules made up of repeating monomeric subunits linked by covalent bonds (Wen et al., 2018). Based on their origin, biopolymers are divided into natural and synthetic classes (Taghipour-Sabzevar et al., 2019). The advantages and disadvantages of these biopolymers are taken into consideration during selection for the development of a drug delivery system.

#### Synthetic Biopolymers

Synthetic biopolymers can be derived from natural polymers or chemically synthesized. They have attracted much attention because of their stability, flexibility, low immunogenicity, and biodegradability. Since they resist hydrolysis and can tolerate high temperatures, they can be heat-sterilized without degradation (Rahman and Hasan, 2019). Poly (α-hydroxy acids), polyhydroxyalkanoates (PHAs), poly (lactones), and poly(alkyl cyanoacrylates) (PACA) are the common synthetic biopolymers, among which poly (α-hydroxy acids) are the most employed class of biopolymers for production of PNs. Poly (α-hydroxy acids) are degraded by non-enzymatic hydrolysis of the ester linkage into non-toxic monomers (lactic acid and glycolic acid). Their degradation rate depends on intrinsic properties such as molecular weight, chemical structure and hydrophobicity (Doppalapudi et al., 2016).

Nanoparticles made up of these polymers have been developed for the delivery of various hydrophilic and hydrophobic anti-cancer agents such as doxorubicin, 5-fluorouracil, cisplatin, paclitaxel, and docetaxel (Rafiei and Haddadi, 2017; Ashour et al., 2019; Domínguez-Ríos et al., 2019; Maksimenko et al., 2019; Mittal et al., 2019).

PLG was the first polymer of this class investigated for biomedical application (Doppalapudi et al., 2016). It is synthesized through the polycondensation of glycolic acid or ring opening of glycolide, but it is not a good choice for the formulation of nanocarriers for cancer therapeutics because of its rigidity and rapid degradation (Shukla et al., 2019). PLA, another widely-investigated polymer, can be obtained from the polycondensation of lactic acid (LA) or by the ring opening polymerization of lactide; it exists in two isomeric forms, poly(l-lactic acid) and poly(d-lactic acid) (Fonseca et al., 2015). PLA naturally degrades *in situ* through the hydrolysis of the ester linkage, rendering LA and its short oligomers as the degradation products. Since the products of PLA biodegradation are cleared easily from the body, its use does not induce severe immune responses (Lee et al., 2016; Casalini et al., 2019).

Among polyesters, PLGA is the most widely-used co-polymer for the development of targeted drug delivery systems, and is made up of glycolic acid and lactic acid monomers (Mir et al., 2017; Maity and Chakraborti, 2020). PLGA polymers undergo complete biodegradation in aqueous media and their characteristics can be altered by varying the chemical composition (lactide/glycolide ratio) and the chain length. For example, the degradation rate and the drug-release rate accelerate when the molecular weight of the copolymer is decreased (Molavi et al., 2020). PLGA can be prepared at different lactide/glycolide molar ratios such as 50/50, 65/35, 75/25, and 85/15. Lactide is more hydrophobic than glycolide, so a decrease in the proportion of lactide increases the rate of hydrolytic degradation of the copolymer, with consequent rapid release of the encapsulated drug (Gentile et al., 2014). It has been suggested that the degradation times of 50/50, 75/25 and 85/15 PLGA is 1–2, 4–5, and 5–6 months, respectively (Middleton and Tipton, 1998; Anju et al., 2020).

Biopolymers produced by microorganisms have shown promise as a substitute for the synthetic polymers currently being used in the industry. For instance, PHAs are naturally produced and accumulated as energy/carbon storage material by many bacteria. PHAs have recently gained great attention because of their biocompatibility, biodegradability, thermoplasticity, low toxicity, and availability (Korde and Kandasubramanian, 2020). They are polyesters of various hydroxyalkanoate monomers that can be produced either through the natural bioconversion process or by chemical synthesis via the ring-opening polymerization of β-lactones (Li and Loh, 2017). Poly(hydroxybutyrate) (PHB) is a PHA derivative used in targeted drug delivery due to its prolonged degradation time *in vivo* and its lesser effect on the pH of tissues as compared to the polylactides (Korde and Kandasubramanian, 2020). According to ISO 10993, PHB nanoparticles have been shown to be safe when used on animals (Masood, 2016).

Among polylactone-based polymers, poly(Ɛ-caprolactone) (PCL) is the most studied polymer for anticancer drug development. It is a semicrystalline compound obtained by the ring-opening polymerization of ε-caprolactone (Witt et al., 2019). PCL exhibits slower ester bond hydrolysis at physiological pH and has a less acidic character than poly-hydroxy acids; in addition, the slower degradation rate of PCL prolongs the release of encapsulated drugs (Doppalapudi et al., 2016).

Poly(alkyl cyanoacrylates) (PACA) are another biodegradable polymer class useful for developing nanocarriers. These polymers are mainly degraded through the hydrolysis of the ester bonds of their alkyl chain. The rate of degradation depends on the alkyl chain length: the longer the alkyl chain, the slower the rate. The two resulting products, namely alkyl alcohol and poly (cyano acrylic acid), are both soluble in water (Nicolas and Couvreur, 2009; Doppalapudi et al., 2016). PACAs can retain substantial amounts of drug (Sulheim et al., 2017). Poly(isohexylcyanoacrylate) nanoparticles containing doxorubicin (Livatag®—see section “Livatag®”.) have been proposed as an innovative formulation for human primary liver cancer and have reached phase III of clinical trials (Merle et al., 2017).

#### Natural Biopolymers

Natural biopolymers include animal- or plant-derived proteins and polysaccharides as well as polymers obtained from microbial sources. These are widely used in drug delivery research due to their unique properties such as abundance in nature, biodegradability, biocompatibility, and low toxicity (Eroglu et al., 2017; Gálisová et al., 2020). However, they can be immunogenic and often require chemical modification before being used for the development of nanoparticles (Karlsson et al., 2018).

### *Animal-Based Biopolymers*

Natural biopolymers of animal origin used for the development of pharmaceutical formulations include albumin, gelatin, hyaluronic acid, and chitosan (Ventura et al., 2011; An and Zhang, 2017; Iannone et al., 2017; Yasmin et al., 2017).

Albumin (MW ∼65–70 kDa) is an endogenous blood protein. Both human serum albumin and bovine serum albumin are used to produce nanosystems for anticancer therapy. They have similar physicochemical properties and produce nanoparticles having similar characteristics (An and Zhang, 2017). Albumin has been used as a nanocarrier for antitumor compounds because of its long biological half-life, which improves the pharmacokinetic properties of the encapsulated drugs and allows the EPR effect to be taken advantage of for increased accumulation in tumor tissues (Karimi et al., 2016; Hoogenboezem and Duvall, 2018). One of the most important formulations of intravenous paclitaxel used in clinical practice is made up of albumin nanoparticles (Abraxane®—see section “Abraxane or Nab-Paclitaxel”).

Gelatin is a heterogeneous mixture of polypeptides derived from the partial hydrolysis of animal collagen (Karlsson et al., 2018). From this process, two types (A or B) of gelatin are obtained. Type B gelatin has been shown to produce nanoparticles with better properties than type A (Yasmin et al., 2017). Gelatin is enzymatically degraded into its aminoacids as a function of several parameters such as pH, temperature or concentration (Sahoo et al., 2015). In general, gelatin is cheap and readily available and could be easily modified to carry targeting moieties; at the same time, gelatin cross-linking can be controlled to alter the drug-relase properties of resultant nanoparticles (Elzoghby et al., 2017). Gelatin nanoparticles have also been investigated for delivery of genetic material (Coester et al., 2000; Magadala and Amiji, 2008). Although gelatin nanoparticles exhibit low toxicity and efficient cellular uptake in cancer cells, the use of gelatins of animal origin carries the risk of contamination with transmissible infection. This drawback could be overcome with the use of recombinant human gelatin, but its widespread use is limited because of the expensive production processes.

Hyaluronic acid (HA) and its derivatives have been employed for biomedical and pharmaceutical applications, in particular for target-specific and long-acting delivery of anticancer agents. HA is a mucopolysaccharide consisting of d-glucuronic acid and N-acetylglucosamine linked together through alternating β-1,4 and β-1,3 glycosidic bonds. It is present in the extracellular matrix and intracellular domain of all living organisms (Tripodo et al., 2015). HA undergoes degradation in the biological environment through hyaluronidases that hydrolyze the β-1,4-glycosidic bonds. The resulting oligosaccharides are degraded by β-d-glucuronidase and β-M-acetyl-hexosaminidase enzymes (Chen et al., 2019a). HA is rapidly cleared from the body, but the linkage of aminoacids to the carboxyl or hydroxyl groups of its chain enhances its blood circulation time (Taghipour-Sabzevar et al., 2019). A close association was found between HA receptor expression and malignant tumor progression. HA receptors (especially CD44, hyaluronan-mediated motility receptor RHAMM, lymphatic vessel endothelial hyaluronic acid receptor 1 LYVE1) are activated in cancer cells to promote cell infiltration and tumor malignancy (Lokeshwar et al., 2014; Lee et al., 2020). HA promotes the uptake of nanoparticles by binding to these receptors (Chiesa et al., 2018; Rho et al., 2018). Another strategy to improve drug delivery and enhance the circulation time of nanoparticles is to covalently conjugate HA onto the surfaces of the nanoparticles (Edelman et al., 2017; Cosco et al., 2019).

### *Plant-Based Biopolymers*

Plant-derived polymers occur abundantly in nature and generally exhibit less immunogenicity than polymers of animal origin. Although cellulose, starch, soy protein, and zein, are the most widely used plant-derived polymers, numerous other plant-based polymers have been studied and have shown excellent results in drug delivery research (George and Suchithra, 2019; Iravani and Varma, 2019; Gagliardi et al., 2020a, 2020b).

Cellulose, the most abundant biopolymer in the world, is a linear homopolymer of 1,4-β glycoside-linked d-glucopyranose (Fattahi Meyabadi et al., 2014; Halib et al., 2017). It is suitable for the development of nanoparticles because it causes no immune-response and its highly hydrophilic structure suppresses opsonization, which is an important phase of phagocytic clearance of nanoparticles. Thus, it prolongs the residence time of hydrophobic drugs in the blood stream and enables accumulation in the target tissue (Varan et al., 2019).

Starch is also a highly available plant-based polysaccharide. All plants synthesize starch as an energy reserve, although this is more common in tuberous plants (potatoes) and cereals (corn, beans, wheat, rice, etc.) (Alcázar-Alay and Meireles, 2015). It is metabolized by amylases and glucosidases into glucose units (Elvira et al., 2002). The advantages of this edible polysaccharide in a controlled release field are the improvement of drug solubility and stability, the reduction of toxicity and side effects, and an excellent biocompatibility and storage capacity (George et al., 2019).

Soy protein nanoparticles have also been investigated for the development of nanoparticles. Like cellulose and starch, it is also a highly-abundant and low-cost material. The amino acid composition of soy proteins (polar, non-polar, and charged amino acids, such as glutamate, aspartate and leucine) promotes the substantial entrapment efficiency of hydrophobic drugs; its solubility characteristics in aqueous environments can be exploited for different administration routes (DeFrates et al., 2018; Voci et al., 2020).

Zein is a low molecular weight protein (∼20 kDa) derived from the cytoplasm of corn cell endosperm (DeFrates et al., 2018; Gagliardi et al., 2019; Gagliardi et al., 2021). It is considered to be a promising biomaterial to obtain nanocarriers containing hydrophobic compounds because it is insoluble in water, except in the presence of alcohol, urea, alkali, and anionic detergents (Elzoghby et al., 2017; Tran et al., 2019).

### *Microbial Biopolymers*

In the previous section, PHAs obtained from microbes was discussed, but they are not the only polymers derived from the microbial world. Many exopolysaccharides originating from microorganisms have also been explored as nanocarriers.

Sulfated polysaccharides are amply exploited in nanotechnology because of their unique physicochemical properties such as noteworthy stability, biodegradability, biocompatibility, and fluid dynamics (Raveendran et al., 2013). Halomonas maura is a bacterium that produces a highly sulfated exopolysaccharide called mauran. Mauran is made up of repetitive units of mannose, galactose, glucose and glucuronic acid and has a high content of sulfate and uronic acid which confer immunomodulatory and antiproliferative effects on human cancer cells (Arias et al., 2003). This high molecular weight polymer has exceptional rheological properties, and exhibits thixotropic behavior. It is also highly resistant to extreme temperatures, freeze-thaw cycles, pHs, and salt-concentrations (Llamas et al., 2006). Raveendran, et al. produced 120 nm nanofibers of mauran and poly(vinyl alcohol) and found them to be an excellent biomaterial for the migration, proliferation and differentiation of mammalian cells (Raveendran et al., 2013). The same group also reported composite nanoparticles of mauran and chitosan for delivery of 5-fluorouracil to glioma and breast adenocarcinoma cancer cells (Raveendran et al., 2015).

### *Biolpolymers From Marine Organisms*

Marine organisms are another rich source of polymers for medical applications. Marine biopolymers such as fucoidan, alginate, carrageenan, and chitosan are renewable, stable, nontoxic polymers that can be potentially harvested at low costs (Manivasagan et al., 2017). Fucoidan, alginate, and carrageenan are obtained from seaweed, whereas microbial chitosan can be isolated from marine crustaceans.

Chitosan is the most widely used cationic polysaccharide approved by the FDA for drug delivery purposes due to its low toxicity, non-immunogenic behavior, and significant compatibility with tissues and cells (George et al., 2019). In addition, it is the only natural positive polysaccharide and can form stable complexes with negative compounds, making it a good candidate for drug encapsulation and controlled release (Yang et al., 2014). Chitosan is a polymer made up of glucosamine and N-acetyl-glucosamine and is mainly obtained from the hard outer skeleton of shellfish, including crab, lobster, and shrimp. It can self-assemble into nanostructures that can penetrate the tight junctions between endothelial cells because of its bio-adhesive properties (Safdar et al., 2019). Chitosan-based nanoparticles are degraded by different enzymes, such as lysozymes, chitosanase, cellulases, lipases and pectinases (Taghipour-Sabzevar et al., 2019). Interestingly, chitosan can also be obtained from microbial sources (Kaur et al., 2012; Amer and Ibrahim, 2019), but this development is still in its infancy.

Fucoidan, which has been recently studied in anticancer nanomedicine, is a sulfated polysaccharide extracted from brown seaweed, principally made up of l-fucopyranose units and sulfated ester groups (Wu et al., 2016). Many studies have reported that fucoidan carries on antitumor activity against a wide variety of human tumors as a consequence of its interaction with P-selectin, a molecule expressed on cancer cells that promotes metastatsis (Shamay et al., 2016; Lu et al., 2017).

Alginate is an anionic linear polymer derived from marine brown algae. It consists of β-d-mannuronic acid and α-l guluronic acid residues linked by 1,4-glycosidic bonds (Venkatesan et al., 2016; Karlsson et al., 2018). These linkages are sensitive to both acid hydrolysis and alkaline β-elimination. In humans the polymer dissolves in surrounding physiological media because of the absence of specific digestive enzymes (Rottensteiner et al., 2014). Its low cost and capacity to interact with various bioactives led to its use in the development of various nanosystems (Joye and McClements, 2014).

Carrageenan is another sulphated polysaccharide, which carries a high negative charge. It can be extracted from different species of seaweed and it is characterized by alternate units of d-galactose and 3,6-anhydrogalactose linked by α-1,3 and β-1,4 glycosidic linkages (Manivasagan et al., 2017). Despite its potential, very few authentic studies exist on the use of carrageenan to produce nanoparticles for anticancer drugs. However, carrageenan has been reported for prolonged drug release in mucosal/epithelial tissues (Kianfar et al., 2013; George et al., 2019).

## Methods of Preparation for Polymeric Nanoparticles and the Role of Surfactants

PNs have been developed with the aim of encapsulating hydrophilic and hydrophobic molecules, such as salts, proteins, and high-molecular-weight DNA or antisense nucleic acids (Cosco et al., 2014; Cosco et al., 2015; Lombardo et al., 2018). A variety of drug classes can be delivered by nanoparticles such as anticancer (Cosco et al., 2011), antifungal (Carraro et al., 2016), anti-inflammatory (Gadde et al., 2014), and anti-leishmanial drugs (Palma et al., 2018). In general, encapsulation favors prolonged and/or controlled release of a drug (Pagels and Prud’homme, 2015), and there is growing interest in nanoparticles for the targeted delivery of entrapped compounds to specific organs or cells (Danhier et al., 2012). The drugs encapsulated in PNs are released by means of diffusion through the polymeric network, erosion of the matrix material, hydrostatic swelling, or by a combination of these mechanisms. A variety of methods has been used to efficiently encapsulate drugs in PNs. The technical choice is dependent on the nature of the polymer selected, desired physicochemical features of the final formulation, and ease and expense associated with the method (Crucho and Barros, 2017).

The most important preparation approaches for PNs are emulsification and solvent evaporation (Yoneki et al., 2015), nanoprecipitation (Rivas et al., 2017; Maggisano et al., 2020), the supercritical anti-solvent method (Kalani and Yunus, 2012), and salting-out (Mendoza-Muñoz et al., 2012). The emulsification and solvent evaporation/extraction technique is the most used method for small and moderate-scale manufacturing of PNs. It is based on the dissolution of polymer in an organic solvent, adding the organic-phase to the water-phase containing stabilizers and surfactants, then emulsification followed by the evaporation of a slowly-boiling organic solvent. Chloroform, dichloromethane, and ethyl acetate are commonly used as organic solvents. Both single oil-in-water (o/w) emuslification (Guo et al., 2015) and double water-in-oil/in-water (w/o/w) emulsification (Cosco et al., 2015) methods are used to obtain an emulsion via high-speed homogenization or ultrasonication. The evaporation of an organic solvent is accomplished by applying heat and vacuum. Spray-drying is the method of choice for getting rid of organic solvents during large-scale production of heat-sensitive PNs (Ozeki and Tagami, 2014).

In the nanoprecipitation method, a water-miscible organic phase is added drop-by-drop into an aqueous phase with or without a stabilizer/surfactant (Rivas et al., 2017). The polymer is deposited at the interface following the displacement of a nonaqueous solvent (for example, acetone) from the solution (Fessi et al., 1989). Traditionally, this easily reproducible technique has been mostly employed for the encapsulation of hydrophobic drug molecules. The nanoprecipitation technique was found to be more efficient than the emulsification method for encapsulating cucurbitacin in PNs consisting of PLGA (Alshamsan, 2014), possibly by preventing the loss of drugs during the emulsification process and increasing entrapment in the polymer matrix. Recently, Salatin, et al. reported the development of rivastigmine-Eudragit RL nanoparticles using the nanoprecipitation method; the entrapment efficiency reached 38% and sustained drug release was observed (Salatin et al., 2017).

The supercritical anti-solvent method is another way to prepare PNs under mild operating conditions. In particular, a polymeric solution is sprayed as tiny droplets into a high-pressure vessel containing an anti-solvent liquid such as CO_2_. The rapid diffusion of CO_2_ into solute favors the formation of nanoparticles. The salting-out method, on the other hand, is based on the addition of a high concentrations of salts (electrolytes such as magnesium chloride, calcium chloride and magnesium acetate) or saccharides (non-electrolyte) to a polymeric solution that induces the appearance of a coacervate; they can be also obtained by the modulation of temperature and pH (Masood, 2016).

Various parameters, such as the molecular weight of the polymers, the presence of cryoprotectants and stabilizing agents, etc. play an important role in the development of PNs (George et al., 2019). The modulation of the polymer length, which is obtained during the synthesis of the polymer, allows one to predict or obtain the desired physico-chemical properties of the system (Wen et al., 2018). For example, the degradation rate of polymers is related to their molecular weight: polymers with low molecular weight degrade faster than polymers with high molecular weight (Kamaly et al., 2016). The rate of polymer degradation also influences the release rate of the encapsulated drugs from the polymer matrix (Crucho and Barros, 2017).

In general, nanoparticles are thermodynamically unstable and attract each other through van der Waals forces in order to decrease their considerable surface energy (Madkour et al., 2019). Therefore, resistance against aggregation is desirable in order to obtain a long shelf life for polymeric nanoparticles. Electrostatic and steric stabilization are the two mechanisms through which nanoparticles are stabilized (Morozova et al., 2019). The former is based on the mutual repulsion of similar electrical charges and depends on the balance of forces between charged surfaces and various interfaces, namely, the attracting van der Waals forces (resulting from dipole-dipole interactions) and the repulsive electrostatic forces of the electrical double layers surrounding the particles in the medium (Morozova et al., 2019). Steric stabilization, on the other hand, is a protective barrier provided by the adsorption or conjugation of polymers or surfactants onto the surfaces of the nanoparticles (Mo et al., 2016).

The use of surfactants is a well-known and widely-employed approach to stabilize polymeric nanoparticles (Heinz et al., 2017). Surfactants stabilize nanaoparticles by reducing the interfacial tension between the solid-liquid phase, which favors interaction between the polymeric system and the suspension medium. Surfactants are classified based on their charge which include the following: (i) anionic (negative charge); (ii) cationic (positive charge); (iii) zwitterionic or amphoteric (charge depends on the pH of the medium), and; (iv) non-ionic (no charge). Generally, non-ionic surfactants are less toxic to the biological membranes than ionic ones and several derivatives have been shown to inhibit the efflux pumps and/or multi-drug-resistance-associated proteins (Rege et al., 2002; Gagliardi et al., 2020c). Examples of common non-ionic surfactants include tweens®, spans®, pluronics®, vitamin E d-α-tocopheryl polyethylene glycol 1000 succinate (vitamin E TPGS), and poly(vinyl alcohol). These surfactants can modulate the size, shape, and surface architecture of polymeric nanoparticles, influencing their therapeutic potential (see Physico-Chemical Properties of Polymeric Nanoparticles) (Heinz et al., 2017).

Pluronics® are water-soluble triblock copolymers made up of a hydrophobic core of polyoxypropylene (POP) between two hydrophilic units of polyoxyethylene (POE) (Giuliano et al., 2020). As GRAS (generally recognized as safe) excipients, they have been widely used in the development of many pharmaceutical formulations (Akash and Rehman, 2015; Bodratti and Alexandridis, 2018; Giuliano et al., 2019). Pluronics® are also referred as “functional excipients” as they carry on important and very useful biological activities (Kabanov et al., 2003; Batrakova and Kabanov, 2008; Giuliano et al., 2018). For instance, pluronics® are known for their attractive ability to sensitize MDR tumor cells toward chemotherapy and reduce cancer stem cell population by depleting intracellular ATP, inactivating permeability-glycoprotein (Pgp)-mediated drug efflux, and rendering cells pro-apoptotic (Waghray and Zhang, 2018; Khaliq et al., 2019). Moreover, pluronics can stimulate the release of cytochrome C and increase the levels of cytosolic reactive oxygen species (Minko et al., 2005).

However, pluronics are not the only surfactants to exhibit anti-MDR effects (Livney and Assaraf, 2013). Tween® 20, Tween® 80, Myrj® 52, and Brij® 30 inhibit protein kinase C (PKC) activity, modulate Pgp-mediated drug efflux, and decrease the apical efflux of the anthracycline epirubicin across human intestinal epithelial (Caco-2) cells (Komarov et al., 1996; Lo, 2003; Tagami et al., 2011; Veeravalli et al., 2020). Tween 80 has been employed as a surfactant in brain tumor targeting (Wen et al., 2018). It enhances the delivery of the active compounds across the blood-brain barrier (BBB) by promoting the adsorption of apolipoprotein E (ApoE) onto the particle surfaces. This enables transcytosis across the BBB through interaction with the low-density lipoprotein receptor-related protein (LRP) expressed on the brain capillary endothelium (Kreuter, 2014; Li et al., 2018; Tosi et al., 2020).

Vitamin E TPGS also inhibits Pgp and enhances drug encapsulation, cellular uptake, therapeutic efficacy, and oral bioavailability of nanocarriers (Guo et al., 2013; Sun et al., 2014; Dong et al., 2016; Choudhury et al., 2017; Peng et al., 2018).

### Physico-chemical Properties of Polymeric Nanoparticles

**FIGURE 3:**
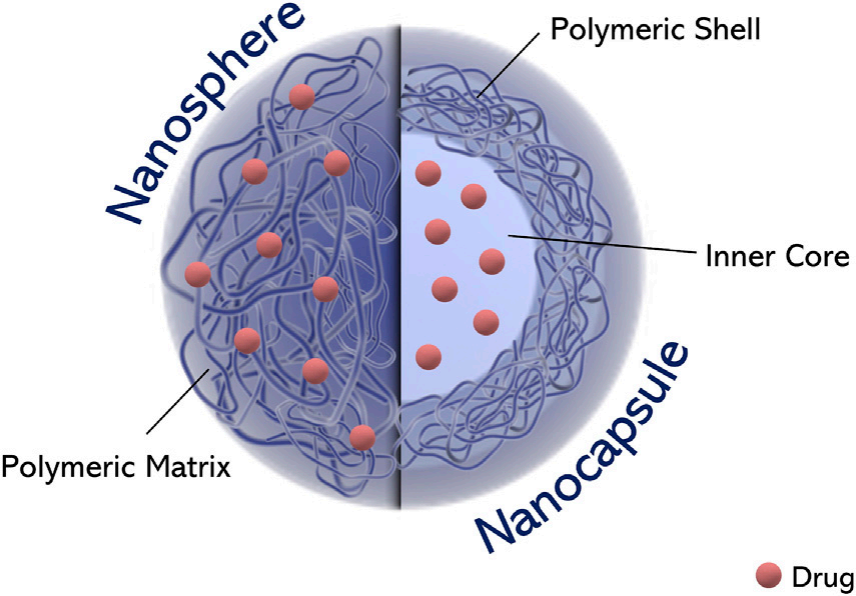
Schematic representation of polymeric nanoparticles as a function of their morphology.

Depending on the processes used for the preparation of polymeric nanoparicles, these can be either nanospheres or nanocapsules (Figure 3). Nanospheres are matrix systems in which the drug is dispersed throughout the structure or adsorbed onto the surface, whereas nanocapsules are systems in which the drug is contained within the core (aqueous or oily) surrounded by a polymeric shell (Paolino et al., 2013; Cosco et al., 2015).

**FIGURE 4:**
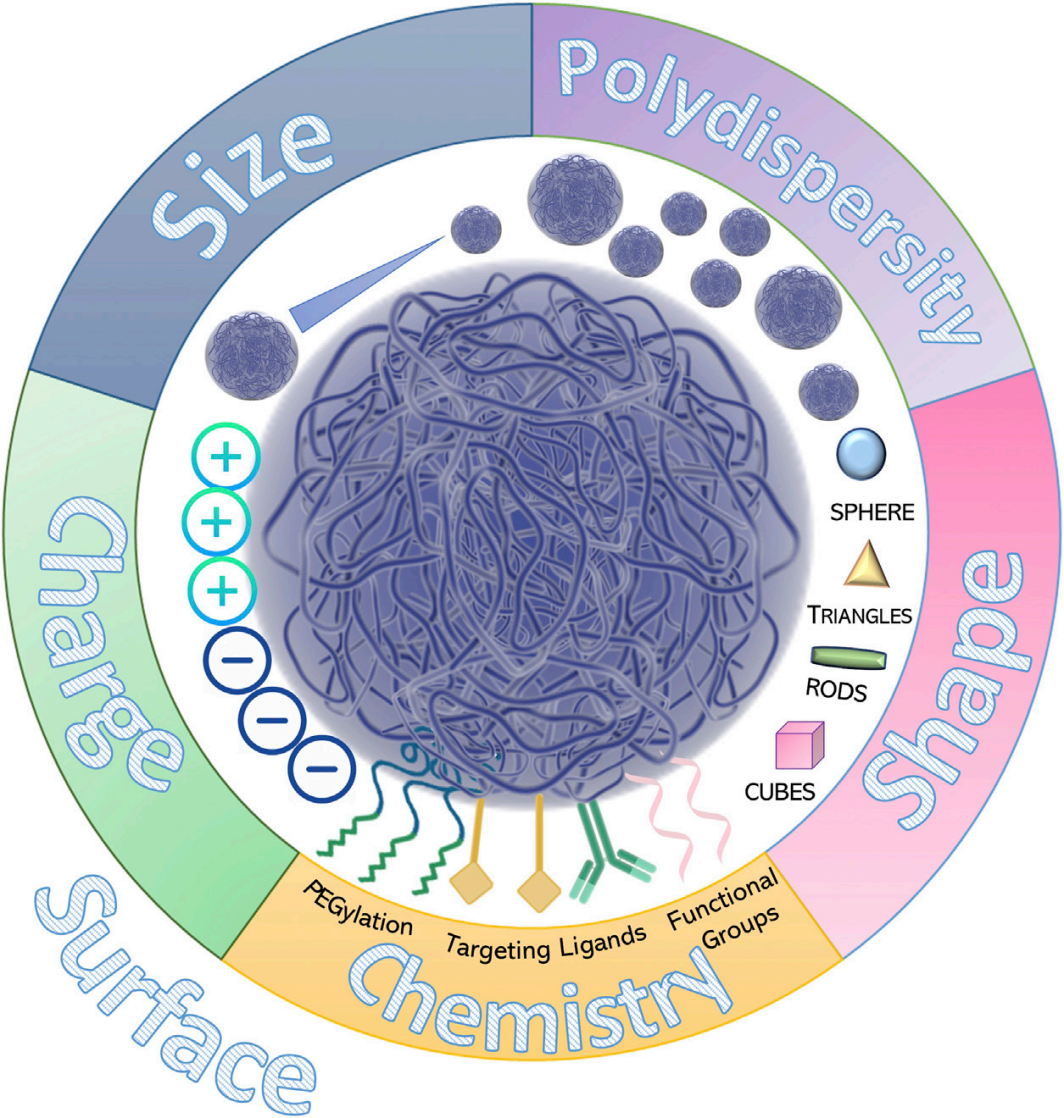
Physico-chemical parameters of polymeric nanoparticles.

The lack of standardized protocols for the characterization of nanosystems has resulted in translational failure of several formulations that were promising for clinical use (D'Mello et al., 2017; Gioria et al., 2018). The physico-chemical properties of nanoparticles, such as size, shape, stability, drug-release profiles and surface characteristics, can all affect their behavior in complex biological environments. At the same time pH and ionic strength of the dispersion medium can influence biodistribution, pharmacological efficacy, and safety of the entrapped drug(s) (Figure 4) (Islam et al., 2017). Indeed, these parameters can significantly change in the biological milieu, due to the adsorption of proteins onto the nanoparticle surface. Recognizing the importance of these physicochemical formulation factors, the European Medical Agency (EMA) and the FDA (Caputo et al., 2019; https://www.fda.gov/downloads/Drugs/GuidanceComplianceRegulatoryInformation/Guidances/UM588857.pdf) both signal the need for the pre-clinical characterization of nanoparticles. In particular, the European Nanomedicine Characterization Laboratory (EUNCL) and the US National Cancer Institute Nanotechnology Characterization Laboratory (NCI-NCL) have developed multiple standard operating procedures for nanomaterial assessment, establishing mean size and polydispersity index as the critical quality attributes of a nanoparticle formulation (Gioria et al., 2018).

### *Particle Size*

The size of nanoparticles used as drug delivery systems should be large enough (diameter of ∼100 nm) to prevent their rapid escape from blood capillaries and renal filtration, but small enough to avoid mononuclear phagocyte system (MPS) clearance (Yang et al., 2016). Several techniques are used to evaluate the mean diameter and size distribution of nanoparticles which include laser scattering (dynamic or static light scattering, laser diffraction), field flow fractionation (FFF), electron microscopy (EM), centrifugation (analytical ultracentrifugation and centrifugal particle sedimentation), tunable resistive pulse sensing (TRPS), and particle tracking analysis (PTA) (Caputo et al., 2019). While many of these are still being perfected (Halamoda-Kenzaoui et al., 2019), dynamic light scattering (DLS) is the most common sizing technique. Even though DLS is characterized by a relatively low resolution, it is highly suitable for the assessment of sample integrity and stability during the initial screening of nanoparticles. However, a combination of multiple high-resolution measurements is often required to demonstrate particle size and size distribution in complex biological media.

Interaction with a biomaterial could favor the formation of aggregates/particles of different mean sizes, leading to significant differences in cell uptake and distribution, toxic effects, and fate within the cell. Any change in size will also impact the pharmacokinetic profile of nanoparticles, alter localization in tissue compartments, and result in unintended interaction with other biological substrates and receptors. For instance, it has been demonstrated that renal filtration and nonspecific uptake by the MPS are dependent on the particle size (Scheinberg et al., 2010). The size and the surface chemistry of NPs affect the opsonization as a consequence of the curvature of systems (Hu et al., 2018). The diameter of particles influences their distribution and adhesion in blood vessels, lungs, and the gastro-intestinal tract. Nanoparticles smaller than 100 nm leave the blood vessels through endothelial fenestrations, whereas microparticles are uptaken by Kupffer cells in the liver or physically entrapped in the capillary beds. Moreover, nanoparticles below 200 nm can be internalized through the clathrin-mediated pathway, while nanoparticles of over 500 nm can be taken up through he caveolae-mediated pathway (Di Marzio et al., 2016).

### *Particle Shape*

In addition to particle size, the shape of nanoparticles is also an important parameter because it affects their pharmacology and functions (Truong et al., 2015). Whereas spherical nanoparticles are the most desired and versatile types with high surface-to-volume ratio and peculiar optical properties, asymmetrical and non-spherical polymeric nanosystems have also been of interest in tissue engineering, immune-engineering, and for theranostic applications (Banik et al., 2016). Because of isometry, spherical particles have better cellular uptake independently of the way they are presented on the cell surface, but in the case of rod-like systems, the uptake is best when they perpendicularly interact with biological surfaces (Stylianopoulos and Jain, 2015).

### *Particle Surface*

Surface characteristics contribute to the solubility of particles, aggregation features, ability to bypass biological barriers, biocompatibility, and targeting properties. The majority of nanoparticles used as drug delivery systems have a hydrophilic surface which is able to favorably interact with the aqueous environment of biological systems. Indeed, a common strategy for avoiding the MPS uptake of nanomaterials is to introduce neutral hydrophilic polymers in order to decrease the opsonization and hence macrophageal phagocytosis. The use of polyethylene glycol (PEG) or poly(ethylene oxide) (PEO) to coat nanoparticles is a prime example of this strategy (Hu et al., 2018). The hydration layer formed by PEG chains around the nanoparticles sterically precludes their interaction with other nanoparticles as well as blood components (Yang and Lai, 2015). In addition, the significant conformational freedom provided by the flexibility of PEG makes the interpenetration of many compounds into the PEG corona thermodynamically unfavorable (Suk et al., 2016). Gref and coworkers developed PEGylated PLGA nanoparticles which had a prolonged plasmatic half-life and reduced liver uptake, compared to the non-PEGylated formulation (Gref et al., 1994). This approach has been used to modulate the pharmacokinetic profiles of many active preparations such as liposomal doxorubicin (Doxil) and micellar-paclitaxel (Genexol) (Barenholz, 2012; Stirland et al., 2013).

The biologic behavior of polymeric nanoparticles is also affected by the surface charge or zeta potential (the electrical potential at the hydrodynamic slipping plane of a particle) (Shao et al., 2015). Cationic or anionic particles are more stable and able to avoid non-specific cellular uptake by phagocytes as compared to neutral ones of a similar size (Wang et al., 2010). Cationic nanoparticles are of immense potential as drug delivery systems because of their strong interaction with negatively-charged genetic material and their ability to bind to cell surfaces. They allow loading of genetic materials which cannot cross cell membranes and ensure effective cell uptake through endocytosis (Farshbaf et al., 2018). Thus, polymeric nanoparticles can be non-viral vectors for gene delivery with high transfection efficiency (Cosco et al., 2014, 2015; Wen et al., 2018). Several remarkable review papers focusing on the application of polymeric nanoparticles for gene delivery are available in literature (Kafshdooz et al., 2016; Suk et al., 2016; Young et al., 2016; Huh et al., 2017; Zhou et al., 2017; Lai and Wong, 2018; Shen et al., 2019; Roma-Rodrigues et al., 2020).

The surface of a nanoparticle is also the place for the conjugation of ligands, with the aim of targeting specific receptors of tissues and organs. As will be discussed below, the patterning of surface groups, also defined by geometric arrangement, influences the geometry of ligands in targeting approaches and also the binding of nanoparticles to the receptors expressed on cancer cells (Banerjee et al., 2016). For this reason it is extremely difficult to develop “smart” nanomedicines able to selectively interact with cancer cells.

## Drug Targeting

**FIGURE 5:**
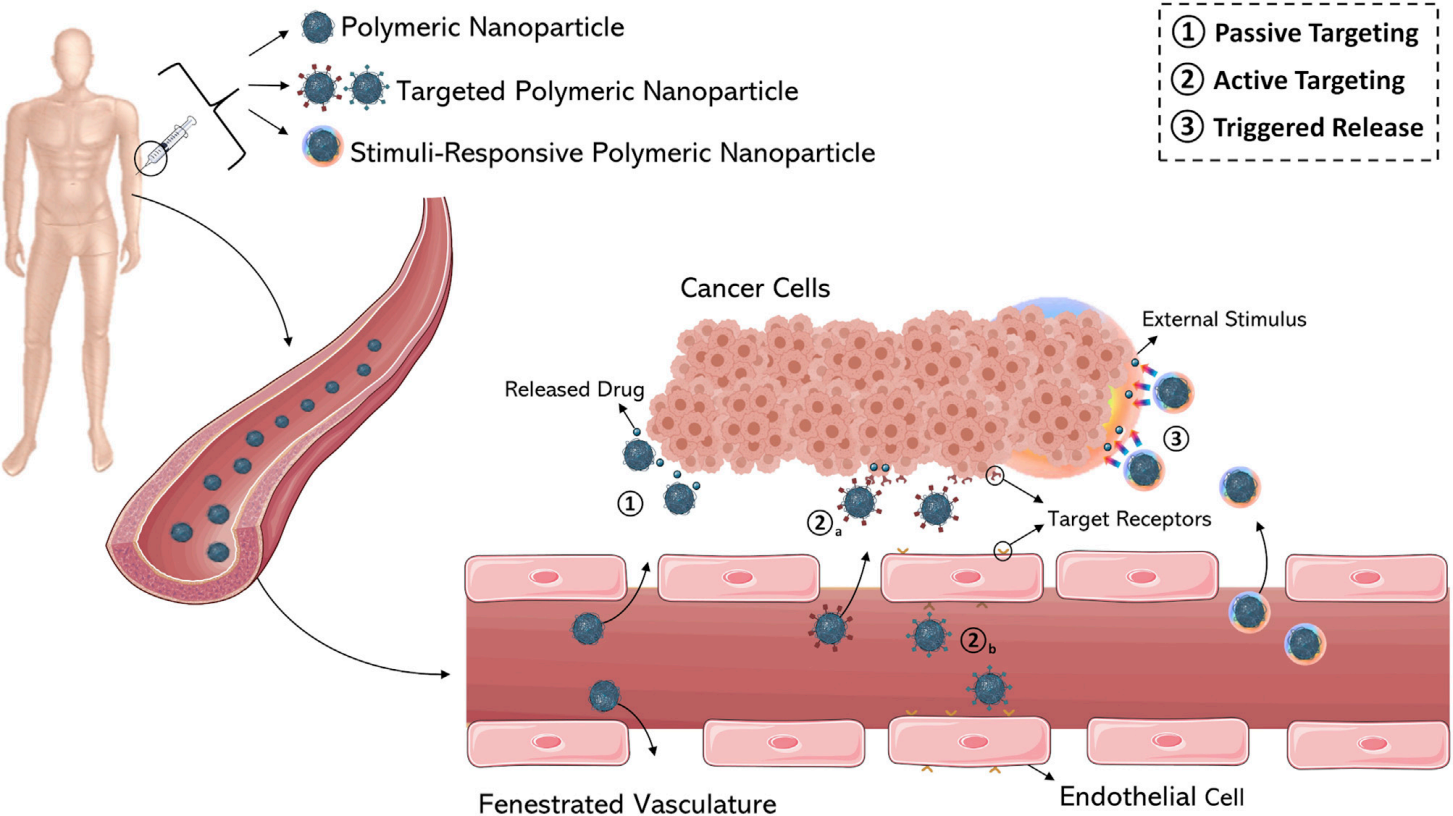
Schematic representation of various drug targeting approaches (1–3). (1) Passive targeting of nanocarriers through fenestrated vasculature of tumor tissue by extravasation. Active targeting of cancer cells (2a) and (2b) tumor endothelium using ligand-modified nanocarriers. (3) Stimuli-responsive nanomedicines able to release the anticancer agent by internal or external triggers. Figure generated from Servier Medical Art.

An important goal in nanomedicine is to combine the unique properties of nanosystems in order to enhance the characteristics of an entrapped drug. As previously discussed, drug delivery within nanoparticles can increase therapeutic efficacy by modulation of the pharmacokinetic and pharmacodynamic profiles exerted by the nanocarrier. These enhancements are partly due to the passive targeting of nanoparticles, which is based on physical interaction between the nanosystems and the tissue microenvironment (blood flow, lymphatic drainage, etc.). Alternatively, nanoparticles can be actively targeted by conjugating tissue-specific ligands (antibodies, peptides, macromolecules, etc.) on the particle surface; specific ligand-receptor interactions increase spatial accumulation of nanoparticles in tissues of interest (Scheinberg et al., 2010) (Figure 5).

### Passive Targeting and Solid Tumors

Passive targeting (Figure 5) exploits the peculiar anatomical and pathological abnormalities of the tumor vasculature which promote the accumulation of polymeric nanoparticles in the perivascular tumor region by convection or passive diffusion (Bazak et al., 2014). Convection refers to the movement of large molecules across large pores. Contrarily, diffusion is defined as a process of molecular transport across the cell membrane along the concentration gradient without consumption of cellular energy and applies mostly to compounds with a low molecular weight. However, diffusion is a more important mechanism of drug accumulation in a tumor mass because convection through the interstitium is poor; the high interstitial pressure of a tumor microenvironment does not allow the convection of drugs. The excessive leakiness of tumor vasculature, characterized by large fenestrations with a mean diameter of 100–800 nm, promotes the localization of nanoparticles into the interstitial space as a consequence of the “enhanced permeability” effect. At the same time, inefficient drainage of the tumor tissue due to the absence or ineffectiveness of lymphatic vessels results in “enhanced retention” of nanoparticles. Together, these two phenomena are known as the “Enhanced Permeability and Retention” (EPR) effect. The EPR effect is the cornerstone in nanoparticle-mediated drug delivery in cancers, especially in solid tumors which are characterized by rapid growth, with the exception of hypovascular tumors such as prostate or pancreatic cancer (Danhier et al., 2012). The size of a nanoparticle impacts the overall manifestation of the EPR effect on nanoparticle accumulation in tumor tissue. Polymeric nanoparticles, micelles, liposomes, and dendrimers of 80–150 nm are retained in the solid tumor tissue, but smaller particles (<20–30 nm) can easily diffuse into other compartments. Therefore, small nanoparticles exhibit poor retention even when they have good permeability (Wang et al., 2010).

The anti-tumor efficacy of many clinically available nanoformulations (Doxil, Caelyx, and Abraxane) can be partially explained on the basis of the passive targeting of the EPR effect. However, recent research has questioned this simplistic model (Mao, 2020). Recently the extravasation of gold nanoparticles of different sizes across various tumor models was investigated by a combination of different imaging techniques and mathematical models. It was observed that most tumor vasculature is continuous and endothelial gaps are much less abundant than previously believed. Accordingly, nanosystems do not extravasate passively via endothelial gaps, but mainly through an active vesicle-mediated transport process called the transcytosis (Sindhwani et al., 2020). Whether this intriguing observation can also be applied to nanoparticles other than gold nanoparticles and whether this phenomenon is universally applicable to all solid tumors remains unclear. As previously described, the composition and the physico-chemical properties (i.e. surface chemistry, shape, charge, etc.) can dramatically affect the *in vivo* fate of nanoparticles. In addition, other parameters such as the tumor type, animal species and the pathological state of patients cannot be excluded from this consideration.

Regardless of the mechanistic bases of passive targeting, the fact remains that a relatively small amount of administered nanoparticles accumulate in tumor tissue. Wihelm and coworkers evaluated the literature of the preceding 10 years and reported that on the average only 0.7% of the administered dose of nanoparticles reaches solid tumors (Wilhelm et al., 2016), so a new generation of nanomedicines with advanced functionalities for the active targeting of tumors is being developed. Nanoparticle systems with stimuli-responsive drug release capabilities are also under development.

#### Influence of Protein Corona on Polymeric Nanoparticles

The most important difficulty in obtaining a successful translation of innovative formulations in clinical practice is the lack of knowledge of their *in vivo* performance at systemic, tissue, and cellular levels (Tang et al., 2017). This large gap between the design of nanosystems and their effective clinical application is primarily due to the partial understanding of the *in vivo* fate of nanomaterials. Once systemically administered, nanoparticles are characterized by a significant modulation of their physico-chemical characteristics. For this reason, a full characterization of nanosystems is a mandatory step in the preformulation phases of novel nanomaterials in order to improve the outcomes in the development of nanomedicines (Corbo et al., 2016). Upon exposure to biological fluids *N of nanoparticles* dynamically and often immediately (<0.5 min) interact with biomolecules such as proteins and lipids through a process defined as opsonization when injected into the blood stream, promoting the formation of the so-called “biocorona” on their surfaces (Cai and Chen, 2019; Lima et al., 2020). The adsorption of opsonins causes their recognition as non-self compounds, promoting their metabolism and elimination by the MPS, leading to a rapid clearance, low efficiency, and high liver accumulation. Notably, opsonins rapidly identify the positively-charged particles with respect to anionic systems (Neagu et al., 2017).

Several types of serum proteins, such as serum albumin, immunoglobulin G, fibrinogen, clustering and apolipoproteins generally present in the biological milieu, can all interact with circulating nanosystems (Lee et al., 2015). It has been reported by several studies that the formation of a protein corona is a dynamic process, induced by the competition of various proteins adsorbed onto the surfaces of nanoparticles and it can be classified as either “soft” or “hard” as a function of the nature of the compounds and interactions involved. Namely, the hard corona presents the first tightly bound layer of proteins over an extended period (many hours), while the soft corona represents a second layer of protein (not directly bound to the nanosystems) facing rapid exchanges for shorter periods (i.e. seconds to minutes) (Lee et al., 2015). A continuous flux of desorption/adsorption of the proteins onto the nanomaterial is controlled by a phenomenon known as the “Vroman effect”, in which precedently adsorbed proteins can be replaced by other proteins with stronger binding affinities until an equilibrium is reached (Vroman, 1962; Frost et al., 2017). This event significantly alters the composition of the protein corona, while keeping the amount of adsorbed proteins relatively constant. In detail, proteins that are present in larger amounts in the biological fluids are the first to be adsorbed onto the nanoparticles during the initial steps of the process (soft corona). These are later replaced by other proteins, less concentrated but having a higher affinity (i.e., slower kinetics), such as lipoproteins, particularly apolipoprotein A-I (hard corona) (Cedervall et al., 2007; Nel et al., 2009). The properties of the nanomaterial (size, shape, surface chemistry and surface charge) as well as the plasma protein characteristics (e.g., human or murine), incubation time, temperature, pH and the physiological state of the plasma (alterations due to disease/medical conditions) greatly affect the adsorption of plasmatic proteins onto the nanosystems (Tenzer et al., 2013). For instance, according to Cedervall et al., human serum albumin (HSA) and fibrinogen showed higher association/dissociation rates than apolipoprotein A-I and other plasma proteins (Cedervall et al., 2007). Moreover, the adsorption of HSA and fibrinogen was more noticeable on hydrophobic particles as compared to hydrophilic ones at the early stages of interaction (Cedervall et al., 2007). It was also reported that hydrophilic particles dramatically reduce the complement activation level (Shreffler et al., 2019), and that the protein corona affects the plasmatic half-time (Bertrand et al., 2017), cell uptake and biodistribution (Chinen et al., 2017), oxidative stress (Jayaram et al., 2017), toxicity (Corbo et al., 2016) and host immune response (Lee et al., 2015) of nanoparticles. This causes a consequent modulation of their *in vivo* behavior and pharmacological outcome.

In this context, Behzadi et al. investigated the potential effect of the biological environment on the release profile of drugs encapsulated in different types of polymeric nanoparticles, demonstrating that the leakage of the active compounds was decreased when the particle surface was decorated with the protein corona (Behzadi et al., 2014). These data demonstrate that the release profile of the entrapped compounds should be properly investigated following the formation of a protein coating in order to predict a real *in vivo* fate of the formulation. Another recent study performed by Alberg et al. evaluated the influence of the protein corona on the physico-chemical properties of three polymeric nanoparticles characterized by long-circulation properties as a consequence of the surface decoration with poly(N-2-hydroxypropylmethacrylamide) (PHPMA), polysarcosine (pSar), and PEG (Alberg et al., 2020). They showed that the mean sizes of the nanosystems was not increased after incubation with human plasma and only a negligible amount of proteins was adsorbed onto the polymeric nanocarriers. This suggests that an intensive corona formation is not a general property of nanoparticles. These results are in contrast with those reported in other investigations (Kokkinopoulou et al., 2017; Weber et al., 2018). However, these findings may explain the slight patient variability of PEGylated polymeric nanocarriers in clinical phase II (CPC634), and evidence the potential of PEG, pSar and PHPMA-based carriers in nanomedicine (Hu et al., 2015; Atrafi et al., 2020).

#### PEGylation and Drawbacks

Polyethylene glycol provides a steric barrier to nanocarriers with the aim of avoiding their interaction with plasma proteins including opsonins and MPS cells, thus prolonging their blood circulation time (Pasut et al., 2015). For this reason, the PEGylation of nanocarriers is a gold standard for improving the therapeutic outcomes with fewer side effects. PEG is the most widely used “stealth polymer” in the drug delivery field, due to its long history of safety in humans and its Generally Regarded as Safe (GRAS) status received from the FDA (Suk et al., 2016). In 1977 Davis and Abuchowsky described for the first time the conjugation of PEG to bovine serum albumin and liver catalases in order to increase the half-life of the proteins with no modulation of their activity (Abuchowsky et al., 1977). Since then, PEGylation has been used in several fields, promoting the development of different formulations characterized by prolonged plasmatic residence time (Veronese and Pasut, 2005). Indeed, the polymer was either directly linked to active compounds or to the surfaces of the nanosystems providing an outstanding stability in biological fluids (Hoang Thi et al., 2020; Sanchez-Cano and Carril, 2020). Namely, it was reported by Klibanov and coworkers that the blood circulation half-life of systemically administered liposomes has been increased by PEGylation from 30 min to 5 h (Klibanov et al., 1990). Later, Gref and coworkers described the first PEGylated PNs, made up of PLGA, evidencing a significant increase in the plasmatic residence time of nanosystems and a reduced liver accumulation with respect to the non-PEGylated systems (Gref et al., 1994). The choice of the more suitable PEG formulation could be very complicated because of the different physico-chemical features of the various derivatives such as the architecture of PEG chains (molecular weight, chain length and several branch arms) that can affect the effectiveness of the polymer as shielding agent. Namely, a study performed by Gref and coworkers investigated the influence of plasma proteins on PEGylated poly(lactic acid) (PLA-PEG) NPs, varying the MW of the hydrophilic polymer (Gref et al., 2000). They demonstrated that the total amount of protein adsorbed onto the surfaces of the nanoparticles significantly decreased when the MW of PEG was a maximum of 5 kDa, but when it was increased to 10, 15, and 20 kDa no additional decrease of adsorbed proteins was observed (Gref et al., 2000). Moreover, it was confirmed by several studies that PEG having a MW of 2 kDa or higher is required to provide a useful steric hindrance on the surfaces of nanosystems in order to decrease protein adsorption and to reduce recognition by the MPS (Bazile et al., 1995; Mosqueira et al., 1999; Fang, 2006; Owens and Peppas, 2006; Perrault and Chan, 2009). The PEG arrangement can also be modulated by the density of the PEG moieties on the particle surface, promoting a mushroom conformation at low polymer densities or a more extended brush state at higher densities (Gulati et al., 2018; Mozar and Chowdhury, 2018).

After several years of clinical use, PEGylation recently showed some potential drawbacks due to the wide application of the polymer. For instance, according to several works, PEG showed a certain non-biodegradability which limits its renal excretion, promoting its accumulation in the liver and in the lisosomes of healthy tissues (Thomas and Weber, 2019). Several hypersensitivity reactions have also been reported (Thomas and Weber, 2019). In addition, there is much experimental evidence that shows a significant level of PEG-related immunogenicity due to antiPEG antibodies detected in the blood of some patients. At the end of the last century, only 0.2% of the population had anti-PEG antibodies, while since 2012 a dramatic increase in the number (up to 25%) has been observed (Garay et al., 2012). This phenomenon suggests that the ample use of PEG-derivatives in various products used daily, especially in cosmetics and food as well as in pharmaceutical formulations, promotes the appearance of related antibodies (Gulati et al., 2018). This can be a significant issue for the colloidal formulations that require repeated systemic administration. Namely some reports evidenced that multiple injections of PEGylated liposomes induced significant immune responses, resulting in a loss of the long circulation half-life of vesicles. This phenomenon is called “accelerated blood clearance” (ABC) and it is characterized by the appearance of anti-PEG IgM, produced by the spleen after the intravenous injection of PEGylated systems that bind the hydrophilic polymer and promote their rapid elimination (Saadati et al., 2013). The ABC phenomenon can compromise the therapeutic efficacy of a drug encapsulated in PEG-coated nanosystems following repeated administrations, modifying the distribution of the compound in tissues. Nevertheless, this effect has also been obtained using PEG-microemulsions, polymeric micelles, nanoparticles and PEGylated proteins (Baumann et al., 2014; Suk et al., 2016). It has been reported that many factors influence the ABC phenomenon, such as the injected dosage, the physico-chemical properties of PEGylated systems, the frequency of use and the animal species. Researchers have employed different animal models to study the ABC phenomenon, including rhesus monkeys, rats, mice and rabbits, but there are various incongruencies with the results (Abu Lila et al., 2013).

However, additional studies of PEG immunogenicity with PNs will be necessary, because many of the experimental works are focused on the systemic immunogenicity of liposomes, which show different physico-chemical features with respect to the polymeric nanosystems. These drawbacks as well as related solutions, have been accurately described in other reviews (Suk et al., 2016; Shreffler et al., 2019). Potential PEG alternatives and the influence of PNs on the stimulation of the immune systems are reported in Supplementary Materials.

### Active Targeting

The active targeting approach is based on the conjugation/integration/adsorption of a ligand to the surface of a nanocarrier with the aim of promoting its interaction with overexpressed receptors specifically in tumor tissue while minimizing interaction with healthy cells (Figure 5). Small molecules such as folic acid and carbohydrates, or macromolecules such as peptides, proteins antibodies, aptamers, and oligonucleotides have been used for these purposes (Wicki et al., 2015). Several anticancer therapeutics, grouped under the name “ligand-targeted therapeutics”, such as trastuzumab (anti-ERBB2, Herceptin®), bevacizumab (anti-VEGF, Avastin®) etaracizumab, and a humanized anti-αvβ3 antibody (Abegrin), have been conjugated onto the surfaces of drug delivery systems in order to promote their accumulation in specific body compartments (Danhier et al., 2010). The level, selectivity, and homogeneity of expression of the target are important factors in the selection of the ligand-target system for active targeting. An overview of the two principal targets (the surfaces of cancer cells and endothelial tumor cells) is provided in supplementary material. A full discussion of this topic is beyond the scope of this review; however, several excellent review articles have been recently published (Mukherjee et al., 2013; Bazak et al., 2014; Zhong et al., 2014; Choudhury et al., 2019; Das et al., 2019; Molavipordanjani and Hosseinimehr, 2019; Raj et al., 2019).

### Stimuli-Sensitive PNs and Trigger Release

In response to physical, chemical, or biological triggers, stimuli-responsive systems promote the release of drugs as a consequence of the structural modulation of the materials (Figure 5). Triggers can be divided into internal stimuli (patho-physiological/patho-chemical condition) which include changes in pH, redox, ionic strength, and shear stress in the target tissues (Li et al., 2013; Lim et al., 2018) and external stimuli (physical) such as temperature, light, ultrasound, magnetic force, and electric fields (Cheng et al., 2013; Liu et al., 2019; Qin et al., 2020).

Several studies in literature demonstrate that the acidification of the tumor microenvironment facilitates the pH-sensitive PNs to release the entrapped drugs into the neoplastic tissue. Lee et al. developed poly(l-histidine)-block-PEG (PbAE) core shell nanoparticles able to release the extrapped doxorubicin at a pH below 7.4 (Lee et al., 2003). This is mainly due to the intrinsic features of PbAE which enhances drug leakage upon exposure to an acid pH; in fact, the unprotonated polymer is insoluble at the physiological pH (7.4) but becomes instantly soluble in aqueous media when the pH of the solution is below 6.5 (Lynn et al., 2001). For this reason, these polymers could be very useful for the delivery of therapeutic agents in solid tumors. Moreover, according to You and August, mild physiological changes in pH ranges between 0.2 and 0.6, could effectively trigger the pH-sensitive poly (N, N-dimethylaminoethyl methacrylate (DMAEMA)/2-hydroxyethyl methacrylate (HEMA) PNs (You and Oupický, 2007; Fang et al., 2015).

Shen et al. developed a novel pH-sensitive delivery nanosystem made up of polymethacrylate grafted poly(amidoamine) modified by folate-PEGylation in order to enhance tumor selectivity and to promote the release of the entrapped drug at an acid pH (Shen et al., 2012).

Ling et al. prepared multifunctional PNs made up of iron oxide and pH-responsive ligands which can target tumors via surface-charge switching, and can be disassembled into a highly active state that ‘turns on’ MR contrast, photodynamic and therapeutic fluorescence activity to selectively kill cancer cells in mice (Ling et al., 2014).

The hypoxic area of tumors rich in reductive agents is another type of microenvironment useful for a triggered drug release by means of disulfide bonds containing PNs (Fleige et al., 2012). In fact, increased glutathione levels lead to a cleavage of disulfide bonds and hence induce drug leakage from the nanosystems into the tumor tissue. External stimuli such as thermal responsiveness, can be applied to a wide spectrum of cancer types, because it relies on local heating to confine the release of drugs, notably improving their clinical application (Crucho, 2015). Moreover, hyperthermia promotes the accumulation of drugs inside the tumor, enhancing the EPR effect of tumor vasculature (Frazier and Ghandehari, 2015). Poly(N-isopropylacrylamide) (PNIPAAm) is a polymer particularly used as a thermosensitive material, because it is characterized by a remarkable phase transition from a water-soluble conformation to an insoluble and hydrophobic aggregate, through a slight increase of temperature. This phenomenon occurs at a low critical solution temperature (LCST) and the phase transition is reversible based on the changes in temperature. This polymer specifically exhibits a LCST of 32°C in water and has been extensively used as a drug carrier (Deptula et al., 2015). Another physical stimulus is light, which includes ultraviolet or near-infrared rays. Light penetrates deeply into the body with increasing wavelengths and the limitation of light absorption by superficial tissues can be easily overcome by special devices such as fiber optic catheters (Jhaveri et al., 2014). Moreover, in order to identify tumor tissue, ultrasound has been used to trigger the release of contrast agents from responsive nanosystems, improving the selectivity of the imaging technique (Joglekar and Trewyn, 2013).

In recent years several studies have shown the ability of stimuli-responsive copolymer-drug bioconjugates to significantly improve the therapeutic index of the active compounds by means of increased accumulation in the tumor sites (Chen et al., 2019b; Zheng et al., 2019; Cai et al., 2020; Guo et al., 2020; Pan et al., 2020; Zhang et al., 2020). For example, Chen and coworkers developed two strategies to achieve this goal: a) through use of N-(1,3-dihydroxypropan-2-yl) methacrylamide with a high molecular weight (MW) linked to doxorubicin by an enzyme-responsive oligopeptide linker and b) by modifying the previously-decribed polymeric derivative by grafting PEG via a disulfide bond. The multistimuli-responsive PEGylated polymeric bioconjugate was characterized by an increased intracellular drug release promoted by the high levels of pH, GSH and cathepsin B in tumor tissues (Chen et al., 2020). The prodrug showed a half-life of 16.9 h, significantly longer than the PEG-free derivative (10.4 h) and DOX (2.7 h), exerting a great antitumor effect and confirming the therapeutic advancement of this approach (Chen et al., 2020).

## Preclinical and Clinical Investigations

### Abraxane or Nab-Paclitaxel

Taxanes, paclitaxel and docetaxel, are cornerstones in the treatment of breast cancer and several other solid tumors (Nicoletti et al., 1993; Geller et al., 2009; Haigentz et al., 2017; Zhu and Chen, 2019). They are highly potent anticancer drugs (Wani et al., 1971), but because of their low aqueous solubility, they require special formulations to enable parenteral administration. In first-generation paclitaxel formulations, polyoxyethylated castor oil (Cremophor-based, Kolliphor® EL, formerly known as cremophor EL) was used to make solubilized-paclitaxel (*sb-*paclitaxel). But cremophor directly contributed to severe and dose-limiting toxicities observed in patients (Brouwer et al., 2000; Chao et al., 2005; Scripture et al., 2005). Abraxane (ABI-007) is a cremophor–free nanoparticulate albumin-bound paclitaxel (*nab*-paclitaxel), which was developed using Celgene’s proprietary nanoparticle albumin-bound (*nab*) technology platform. Abraxane combines paclitaxel with a naturally occurring human albumin protein and exploits endogenous albumin transport pathways, resulting in enhanced transport across endothelial cells. The transcytosis of *nab*-paclitaxel is facilitated by its binding to the gp60 receptor and caveolar transport. In the interstitium, *nab*-paclitaxel binds to the Secreted Protein Acidic and Rich in Cysteine (SPARC), which is overexpressed in the majority of solid tumors, thus achieving enhanced drug targeting and penetration in tumors. Abraxane delivers 49% more paclitaxel to tumors as compared to solvent-based paclitaxel formulations and eliminates solvent-mediated toxicities, such as hypersensitivity reactions (Gradishar et al., 2005). Abraxane has been approved in 41 countries, including the USA, Canada, the European Union and Japan as first-line treatment for metastatic breast cancer. In combination with platinum drugs, it is also used in non-small cell lung cancer patients (Chen et al., 2015; Adrianzen Herrera et al., 2019).

Abraxane consists of paclitaxel stabilized within ∼130 nm albumin nanoparticles in a non-crystalline state. It is used as a colloidal suspension of 5 mg/ml paclitaxel reconstituted in 0.9% saline from a lyophilized formulation of paclitaxel and human serum albumin. In a comparative study, Abraxane was found to possess better physicochemical stability post-reconstitution than two other cremophor-free formulations, namely Genexol and Nanoxel, in which paclitaxel was solubilized in micellar form by an amphiphilic block copolymer methoxy-poly(ethylene glycol)-*block*-poly(d,l-lactide) (Ron et al., 2008). Following intravenous administration, amorphous paclitaxel solubilizes as a smaller albumin-bound paclitaxel complex. It was also associated with pharmacokinetics which were distinct from those of *sb-*paclitaxel (Chen et al., 2014). Apart from a more rapid and broader distribution, *nab*-paclitaxel also exhibited extensive and deep tissue penetration. The rates of the distribution of paclitaxel more than doubled when administered as *nab*-paclitaxel vs. *sb*-paclitaxel (Chen et al., 2014). At the same time, the elimination of paclitaxel was much slower for *nab*-paclitaxel as compared to *sb*-paclitaxel, suggesting that *nab*-paclitaxel allows for an increase in the systemic exposure of paclitaxel (Sonnichsen et al., 1994; Ibrahim et al., 2002). Moreover, unlike *sb*-paclitaxel, *nab*-paclitaxel distributes evenly between the cellular and plasmatic components of blood; *sb*-paclitaxel concentrates largely in plasma and is not readily distributed to blood cells (Lyman et al., 2005). Both *nab*-paclitaxel and *sb*-paclitaxel showed a similar tendency to supress an absolute neutrophil count (Joerger et al., 2007), suggesting that the suppression of hematopoiesis is not altered by the nanoparticulate formulation. Overall, the advent of Abraxane has been a landmark in the therapy of solid tumors using taxanes, and this development has been attributed to a significant improvement in the safety and efficacy of paclitaxel as *nab*-paclitaxel as compared to *sb*-paclitaxel (Vishnu and Roy, 2011).

### BIND-014®

BIND-014 is a PEGylated polylactic acid nanoparticle containing docetaxel conjugated with a small-molecule targeting prostate-specific membrane antigen (PSMA) for prostate cancer. This preparation allows the gradual release of docetaxel upon degradation of the polylactic acid, and the presence of surface PEG enables its escape from the host’s immune response while the PSMA ligand restricts the cytotoxic effect to PSMA-expressing cells (Chang et al., 1999; Rajasekaran et al., 2005). Developed by BIND Therapeutics, Inc (Cambridge, MA), BIND-014 is a targeted nanoparticle of approximately 100 nm. Preclinical studies showed pharmacokinetic properties of BIND-014 that were markedly different from those of *sb*-docetaxel. It exhibited a higher peak concentration (C_max_), a greater area under the curve, and a lower volume of distribution and clearance, indicating that BIND-014 is retained in the plasmatic compartment and releases docetaxel at a slow and controlled rate (Summa et al., 2015). Administration of BIND-014 to animals bearing tumor xenografts was found to result in higher intra-tumoral docetaxel concentrations and increased anti-tumor activity as compared to free docetaxel (Summa et al., 2015). In phase 1 clinical studies, BIND-014 was reported to be well-tolerated as compared to conventional docetaxel (Von Hoff et al., 2016), which led to a phase 2 study called iNSITE 1 (investigation of Sacroiliac Fusion Treatment) in patients with metastatic castration-resistant prostate cancer (Autio et al., 2016). In this phase 2 study, BIND-014 demonstrated a 52.5% 6-week disease control rate for the intention-to-treat population and 70% in the per-protocol population, which exceeded the trial target rate of 65% (Autio et al., 2018). However, a second study (iNSITE 2) in patients with cervical and head and neck cancers reported the poor performance of BIND-014 in reaching study goals. Based on these results and lack of resources to develop and further test this technology, clinical trials were aborted and BIND Therapeutics filed for bankruptcy in 2016.

### Livatag®

Livatag is a nanoparticulate doxorubicin formulation developed by Onexo (Paris, France). The drug is encapsulated within 100–200 nm nanoparticles composed of polyalkylcyanoacrylate, cyclodextrin, and poloxamer (doxorubicin Transdrug) and these were presented as ultra-dispersed colloidal systems for the treatment of primary liver cancer. The success of Livatag depended on its efficacy to treat cancer cells that had become resistant to chemotherapy, with an assumption that the nanoparticle formulation would prevent the rejection of doxorubicin outside the cell. Although doxorubicin is commonly used for the intra-hepatic arterial delivery of chemotherapy for hepatocellular carcinoma (Llovet et al., 2002), free doxorubicin is associated with high morbidity, and its efficacy in tumor regression and overall survival is poor (Lai et al., 1988). Initial studies with Livatag showed that it generated a 12-fold increase in drug exposure within the hepatic tumor tissue as compared to free doxorubicin, without increasing the drug’s exposure in the heart or other vital organs (Bennis et al., 1994). Nanoparticles were taken up by the liver after only a few minutes and this approach appeared to avoid the drug resistance mediated by the rapid efflux of free drug (Bennis et al., 1994). However, Livatag’s clinical trail showed frequent and severe adverse pulmonary events; at the same time, its efficacy was in the same range as that achieved by other existing drugs. Moreover, the trial found no dose-dependent differences between the two Livatag arms, and it failed to meet the primary endpoint in a phase 3 trial in adult patients with unresectable hepatocellular carcinoma. Based on these observations, further development of Livatag was stopped (Merle et al., 2017).

## Conclusions and Perspectives

Despite the fact that PNs have been considered promising formulations for cancer therapy, their successful application is limited by various drawbacks (Kumari et al., 2016). In particular, changes in the physico-chemical properties of nanocarriers (size, surface charge, aggregation, appearance of protein corona) promoted by the components of the blood stream and early drug release in addition to the development of multiple drug resistance by cancer cells, all limit their pharmacological efficacy. Moreover, the toxicity of PNs made up of novel materials, including organic polymers or mixed systems with inorganic materials such as gold, silver oxide and silica are issues for clinical application. The particle size, shape, sedimentation, drug encapsulation efficacy, desired drug release profiles, distribution in the body, circulation and cost are some of the parameters used to select suitable formulations for an efficient cancer targeted drug delivery (Biswas et al., 2013).

However, although many efforts have been made to develop novel targeted nanocarriers, only a few of them are approved for clinical use by the FDA (Barenholz, 2012). This phenomenon could be due to the lack of knowledge on the distribution and accumulation of targeted nanoparticles after oral or intravenous administration and/or to the deficiency of regulatory aspects (e.g., study design and approval challenges) (Kumari et al., 2016). The future of nanomedicine, especially by means of PNs, will improve the efficacy of conventional therapies by exploiting the concept of personalized therapy as a consequence of the opportunity of modulating the various parameters of nanosystems as previously described. For instance, the application of PNs for the combined therapy of tumor (simultaneous delivery of multiple anticancer drugs/combination of conventional chemotherapeutics with other treatment modalities) as well as the delivery of anticancer drugs in association with photosensitizing agents, nucleic acids, antiangiogenic compounds may all better exploit the versatility of the proposed systems and their ability to overcome MDR mechanisms thus increasing the final anticancer effect. The continuous research on PNs in both preclinical and clinical studies will improve the prevention, diagnosis and treatment of cancer.

## Author Contributions

AG, EG, SB, VA, and DC conceived the review and drafted the original manuscript. AG, EG, VE, and MF analyzed the literature data. SB, VA, and DC supervised the study. All authors approved the final manuscript.

## Funding

This paper was financially supported by funds from the Department of Health Sciences, University of Catanzaro “Magna Græcia”.

## Conflict of Interest

The authors declare that the research was conducted in the absence of any commercial or financial relationships that could be construed as a potential conflict of interest.
