# Supplementary material for: Biodegradable Polymeric Nanoparticles for Drug Delivery to Solid Tumors
Source: Front Pharmacol. 2021 Feb 3;12:601626. doi: 10.3389/fphar.2021.601626 (PMC7887387; doi:10.3389/fphar.2021.601626)
Supplement: Supplementary file 1 [file datasheet1.docx]

*Supplementary Material*

## Potential PEG alternatives

A goal of nanomedicine is the development of novel strategies able to decrease or avoid the immunogenicity of PEG-coated nanocarriers with the aim of preserving their *in vivo* performance (Cosco et al, 2017). Even though this review describes the antitumor applications of polymeric nanoparticles, in this section the most used alternatives to PEG have been discussed with the aim of providing useful information for the readers (Table S1). Some reports described the effect of a derivative made up of a poly(methylmetacrilate) backbone linked to 9 side-chain ethylene glycol moieties (POEGMA) on the immune system activation. The antigenicity significantly decreased when the copolymer was administered in patients with respect to to Krystexxa, a FDA-approved PEGylated protein. Moreover, the use of 3 chains of ethylene glycol showed both non-antigenicity and non-specific protein adsorption (Qi et al., 2016, Gulati et al., 2018, Joh et al., 2019). This chemical design could be a useful strategy to combine EG oligomers with other polymer backbones in order to develop an innovative shielding strategy as alternative to linear PEG.

Moreover, much efforts have been made to discover and investigate the potential application of synthetic and natural hydrophilic polymers to be used on patients. They include vinyl polymers, such as poly-acrylamide (PAAm), poly(N,N-dimethyl acrylamide) (PDMA), poly-N-(2-hydroxypropyl)methacrylamide (PHPMA) and poly-vinylpyrrolidone (PVP), poly-glycerols (PGs), and poly(oxazolines) (POX).

Other interesting compounds/derivatives used as coating agents able to increase the plasmatic half-life of a nanosystem are hyaluronic acid (HA), heparin, polisialic acid, poly (aminoacids) and the membranes of red blood cells (RBCs).

#### Hydrophilic polymer-based shielding approaches

PAAm and PDMA are non-ionic polymers available as linear, substituted and branched forms widely used to modulate the pharmacokinetics profiles of several drug delivery systems thanks to their peculiar characteristics of non-immunogenicity, low cost, stability over a wide range of pH (pH 3-11) and ability to decrease the protein interaction (You et al., 2007, Knop et al., 2010; Hadjesfandiari et al., 2018). However, the toxic monomers derived by the polymerization procedure can promote severe neurotoxic effects, limiting their use as PEG alternatives (Erkekoglu et al., 2014). Conversely, the use of PHPMA as a shielding agent revealed a significant efficacy when conjugated with chemotherapeutic drugs (Abbina et al., 2018). Indeed, the formulation of PHPMA-doxorubicin is currently in phase III of clinical trials, due to both the low immunogenicity and low accumulation in liver and spleen (Knop et al., 2010, Ulbrich et al., 2010). Moreover, the PHPMA-paclitaxel derivative is under investigation in clinical phase I/II for the treatment of various carcinoma, evidencing a lack of efficacy in some patients (Knop et al., 2010). These findings suggest that additional studies need to be performed in order to better investigate the properties of PHPMA.

In the last years PGs have been used as potential candidates to confer long circulation properties to various colloidal systems (Suk et al., 2016). The PGs are highly biocompatible, hyperbranched polyols, which showed, long plasmatic half-life (33 h for 106 kDa and 57 h for 540 kDa) and low immunogenicity, but they also evidenced liver and kidney accumulations (Hadjesfandiari et al., 2018). Abu-Lila and co-workers showed that PEG-coated liposomes were less susceptible to ABC phenomenon after repeated administration compared to PEG-coated liposomes, suggesting that the steric hindrance mediated by PG molecules may limit the binding of PG-liposomes and immunoglobulins to splenic B cells, preventing the generation of anti-polymer antibodies (Abu Lila et al., 2013; Mohamed et al., 2019).

Another interesting class of molecules widely used for different pharmaceutical and biomedical applications belongs to the family of poly(oxazolines) (POX) which showed physico-chemical properties comparable with those of PEG (Humphries et al., 2020). The introduction of different residues on the oxazoline ring of the monomer can affect and increase the blood circulation time of nanosystems. POX-coated silica-, gold- and magnetic nanoparticles provided showed interesting stealth properties, but the high cost of synthesis and the difficulty to obtain the FDA approval have been detrimental for the use of the polymer as PEG alternative (Viegas et al., 2011; Abbina et al., 2018; Khutoryanskiy et al., 2018).

Among the vinyl polymers, PVP was the most attractive compound used as PEG alternative due to its broadly use for several years as safe excipient in numerous food, cosmetics and pharmaceutical applications (Liu et al., 2013). It showed a lower degradation under UV or ultrasound irradiation with respect to PEG. Ishihara and coworkers demonstrated that PVP-coated nanoparticles increased their blood circulation time preventing the i ABC phenomenon (Ishihara et al., 2010). However, other experimental investigation evidenced an increase of the absorption of plasmatic proteins on these systems suggesting an unclear immunological behavior of PVP-derivatives. In addition, the toxic and carcinogenic features of free monomers limit the use of this polymer on humans (Zhang et al., 2016).

#### Carbohydrate-based shielding approaches

The use of carbohydrate as shielding materials represents another approach to promote the long circulation properties of nanosystems. Glycosaminoglycans (GAGs) are unbranched, negatively charged polysaccharides which are located at the cell surface and/or extracellular matrix.

A monosaccharide such ad polisialic acid (PSA, a homopolymer of α2,8-linked sialic acid) showed shielding effect of nanosystems by preventing the recognition of nanosystems by the macrophages. A study performed by Wilson and coworkers showed that PSA-coated micelles containing cyclosporine A are characterized by a prolonged half-life and an antitumor effect similar to the free drugs (Wilson et al., 2014). Jung et al. recently showed that doxorubicin-loaded PSA nanoparticles evidenced low toxicity in healthy tissues in mice, and described the biodegradable and biocompatible nature of PSA (Jung et al., 2017).

Among the GAGs, HA is the most endogenous macromolecule used as shielding agent due its characteristics of non-toxicity, biocompatibility, biodegradability, and non-immunogenicity (Dosio et al., 2016). High molecular weight HA maintains cell integrity and water content in the extracellular matrix, while a progressive weight reduction of HA strongly promotes angiogenesis and inflammatory phenomena (Zamboni et al., 2018). Indeed, as reported in the previous section, the polymer has been widely used in drug delivery as PEG alternative as well as targeting ligand against various receptors such as CD44, RHAMM, HARE LYVE-1 (Nascimento et al., 2015; Cosco et al., 2017, 2019). Several investigations have been performed on HA-coated polymeric nanoparticles in order to demonstrate the peculiar stealth effect of this macromolecule (Yadav et al., 2007; Vangara et al., 2013; Huang et al., 2014; Youm et al., 2014; Hu et al., 2015; Park et al., 2015; Cerqueira et al., 2017; Zamboni et al., 2017).

Heparin is another GAG known for its anticoagulant properties and the ability to inhibit angiogenesis and inflammation (Cassinelli et al., 2016). It is also been used as shielding agent of various types of nanoparticles, showing encouraging results due to the long circulation properties exerted by the macromolecule (Jaulin et al., 2000; Socha et al., 2008; Park et al., 2006).

#### Other approaches

A different strategy is based on the use of red blood cell (RBCs) membranes with the aim of avoiding a rapid clearance of nanoparticles. Hu and coworkers developed PLGA nanoparticles coated with RBC membrane, evidencing an increase of in vivo half-life of nanosystems compared to PEGylated carriers (Hu et al., 2011). In another study, the authors showed that this property was due to the presence of both a RBC glycocalyx and a glycosylated transmembrane protein (CD47) on the membrane surface that bind SIRP (signal-regulatory protein) preventing the clearance of particles (Hu et al., 2014). However, the different blood groups or the potential contamination could represent a real challenge to the immune response. For this reason, the use of autologous erythrocytes as coating agents of nanoparticles can be a useful approach (Thomas et al., 2019).

Another class of molecules investigated to bypass the PEG-related issues belongs to the family of poly (aminoacids). Among them, polyglutamic acid (PGA) is one of the most used because it has been approved by the FDA and EMA for food, pharmaceutical and cosmetic application (Obst et al., 2004). The PGA-paclitaxel conjugate (OPAXIO^®^) was the first poly (aminoacid)-based drug included in the phase III of clinical trials for the treatment of various carcinoma. It demonstrated to decrease the immune response with respect to PEG, but a certain complement activation was observed. In fact, it induced a moderate hypersensitivity reaction during the treatment of patients (Suk et al., 2016).

Promising alternatives to PEG are also synthetic zwitterionic derivatives, such as poly(carboxybetaine) (pCB) and poly(sulfobetaine) (pSB), which demonstrated high resistance to nonspecific protein adsorption as well as low immunogenicity. Several experimental studies have been performed demonstrating the efficacy of poly(carboxybetaine) as coating agent of shielding on different types of nanoparticles such as silica (Jia et al., 2009), gold (Yong et al., 2009), iron oxide (Zhang et al., 2010) and PLGA nanosystems (Cao et al., 2010; Cao et al., 2012). They showed a significant serum stability of these formulations and poor immune responses after administration (Amoozar et al., 2013; Hoang Thi et al., 2018).

**Immunological effects for oncological applications**

The great potential advancements in anticancer therapy that can be obtained by means of PNs are unquestionable. Unfortunately, even though they have been developed with the aim of decreasing the side effects of the encapsulated drugs, PNs can also indirectly induce many immune-toxic effects, reducing the pharmacological efficacy of the nanomedicine (Hannon et al., 2019). The reasons of these reactions and the related immune-toxicity are not fully understood because they depend by their composition, physico-chemical properties and administration route (Ilinskaya and Dobrovolskaia, 2013). A strong correlation between the immune-toxicity of PNs and high levels of pro-inflammatory cytokines, activation of stress-related genes, membrane destabilization and interaction with various components of the immune system has been identified (Pandey and Prajapati, 2018).

Endotoxin (a lipopolysaccharide component of the cell walls of gram-negative bacteria) contamination is also an important factor to consider when assessing the immune-toxicity of PNs. Endotoxin can easily contaminate nanomaterials during their production or handling, leading to serious health issues. Therefore, to minimize the risks, the contamination of nanomaterial used for the development of PNs should be avoided (Vetten et al., 2014).

Several scientific papers available in literature described the causes of the immune-toxic effects of PNs and the possible sterilization methods that can be used (Vetten et al., 2014; Dobrovolskaia, 2015; Li and Boraschi, 2016; Agrahari and Agrahari, 2018; Engin and Hayes, 2018; Pandey and Prajapati, 2018; Hannon et al., 2019; Muhammad et al., 2020)

Different approaches have been investigated to decrease the interactions between nanoparticles and the immune system (see sections 6.1.2.). At the same time, engineered PNs with immune-stimulating features can represent a useful tool in anticancer nanomedicine.

It is well known that cancer cells can escape or even suppress the body’s natural immune response, but various components of the immune system play an essential role in protecting humans from neoplastic diseases (Farkona et al., 2016). Cancer immunotherapy focuses on the stimulation of the immune system of the patients to contrast the growth of cancer (Ventola, 2017a). This idea is based on the hypothesis that the immune system can eliminate potentially malignant cells and tumors in a process called “immune surveillance” (Ribatti, 2017). Tumors arise through a combination of genetic and epigenetic factors that enable immortality, but at the same time exhibit on their surface antigens, the tumor-associated antigens (TAAs), which are recognize by the immune system (Pardoll, 2015).

Many immune-stimulating agents have been developed, including adjuvants, cytokines and monoclonal antibodies, and have shown great potential to inhibit the cancer growth (Zhuang et al., 2019). However, their direct administration promotes a rapid degradation and off-target problems, resulting in a low therapeutic and adverse effects such as excessive inflammation, toxicity, and hypersensitivity (Ventola, 2017b).

The encapsulation into PNs is an emerging strategy to improve the efficacy of these immunotherapeutic agents thanks to several benefits compared to their free form such as i) the protection by metabolism and non-specific cellular interactions, ii) the opportunity to obtain a selective delivery to tumor sites and iii) the co-delivery of multiple immune-stimulants and/or antigens (Bertrand et al., 2014; Fang et al., 2015; Dehaini et al., 2016; Zhuang et al., 2017, 2019).

The mean sizes of nanoparticles influence their cell localization: larger nanoparticles (>100 nm) are uptaken by macrophages and dendritic cells of the innate immune system, while smaller nanoparticles (10-100 nm) can easily accumulate in lymph nodes passing through the tight junctions between vascular endothelial cells (Ryan et al., 2014; Jiang et al., 2017). The surface of nanoparticles is another important parameter that can be modulated to obtain an immune-stimulating effect. Indeed, it has been shown that that negatively charged and PEGylated nanosystems are localized in lymph, while positively charged particles are more efficiently uptaken by dendritic cells (Jiang et al., 2017). Moreover, some biomaterials containing hydrophobic domains have been shown intrinsic adjuvant activity. PNs made up of polymers like PLGA or chitosan can stimulate the immune cells without additional agents (Park et al., 2018). Park and Babensee evaluated the influence of different commonly used biomaterials on the maturation of dendritic cells (DCs) (Park and Babensee, 2012). They showed that PLGA and chitosan promoted the DC maturation and induced a significant allostimulation characterized by pro-inflammatory cytokines release, CD44 expression, and endocytic ability (Park and Babensee, 2012).

In summary, nanotechnology can improve the safety and efficacy of immune-modulatory compounds and it is a promising option for cancer therapy that can improve the quality of life of patients.

## Targeting of cancer cells

The active targeting of cancer cells is particularly attractive to increase the intracellular delivery of drugs, such as DNA, siRNA, proteins, salts, etc (Bazak et al., 2015). An important parameter to be evaluated during the conjugation of a ligand on the particle surface is its affinity for a receptor and the cell uptake of systems after ligand/receptor binding (Li et al., 2017) Various receptors have been investigated to target cancer cells such as the transferrin or the folate, glycoproteins, the epidermal growth factor receptor (EGFR) or integrins (Steichen et al., 2013).

The transferrin receptor is a target for cancer therapy involved in iron homeostasis and the regulation of cell growth. The levels of expression of transferrin receptor in cancer cells may be up to 100-fold higher than the average expression of healthy cells (Tortorella et al., 2014).

The folate receptor is overexpressed in many tumors. Folate-grafted nanocarriers are accumulated in cancer cells via receptor-mediated endocytosis as a consequence of the high-affinity interaction between the ligand and its receptor. The alpha isoform of folate receptor is overexpressed on 40% of human cancers. On the contrary, the isoform beta is expressed on activated macrophages and also on the surfaces of hematopoietic malignant cells (Assaraf et al., 2014).

Cancer cells often expose different glycoproteins on their surfaces. Proteins of non-immunological origin like lectins are able to recognize and bind to specific residues of glycoproteins expressed on the cell surface. This approach could be considered as a strategy for colon drug targeting (Danhier et al., 2010).

A family of tyrosine kinase receptors such as the Epidermal Growth Factor Receptor (EGFR) is another class of targets to be used in order to obtain an efficient drug delivery. EGFR is frequently overexpressed in a lot of solid tumors, including colorectal cancer, non-small cell lung cancer and squamous cell carcinoma of the head and neck, as well as ovarian, kidney, pancreatic, and prostate cancers (Prabhu et al., 2017). The activation of this receptor stimulates key processes involved in tumor growth and progression, like proliferation, angiogenesis, invasion and metastasis.

## Targeting the tumor endothelium

The lack of oxygen and nutrients can play a crucial role in the destruction of the endothelium in solid tumors, causing the death of tumor cells. The observation of Judah Folkman in 1971, suggested that the tumor growth might be inhibited avoiding the recruitment of new blood vessels (Folkman et al., 1971). For this reason, the size and the metastatic properties of tumors can be regulated by ligand-targeted nanocarriers, able to bind and to kill angiogenic blood vessels and indirectly the tumor cells. There are numerous advantages deriving from the targeting of the tumor endothelium, including (i) the direct binding of nanocarriers to their receptors after intravenous injection, without unspecific extravasation, (ii) the decrease of resistance compared to cancer cells, due to the genetically stability of endothelial cells (Wang et al., 2017)

The targeting of tumor endothelial cells occurs through the interaction of specific ligands with their receptors, such as:

the vascular endothelial growth factors (VEGF) and their receptors, VEGFR-1 and VEGFR-2 (Shibuya et al., 2013);

the endothelial cell receptor for extracellular matrix proteins, for example αvβ3 integrin that interacts with fibrinogen (fibrin), vibronectin, thrombospondin, osteopontin and fibronectin. The αvβ3 integrin is important in the calcium dependent signaling pathway leading to endothelial cell migration and it is also overexpressed on neovascular endothelialium compared to healthy endothelial cells and the most of normal organs. Cyclic or linear derivatives of RGD (Arg–Gly–Asp) oligopeptides are the most studied peptides which bind to endothelial αvβ3 integrins (Nieberler et al., 2017);

the immunoglobulins such as the vascular cell adhesion molecule-1 (VCAM-1) that is expressed on the surface of endothelial tumor cells and induces the cell to-cell adhesion, a key step in the angiogenesis process. The VCAM-1 is overexpressed in various cancers, including leukemia, lung and breast cancer, melanoma, renal cell carcinoma, gastric cancer and nephroblastoma (Schlesinger et al., 2015);

the zinc dependent endopeptideases, like matrix metalloproteinases (MMPs) playing an essential role in angiogenesis and metastasis, especially in endothelial cell invasion and migration, in the formation of capillary tubes and in the recruitment of accessory cells. Membrane type 1 matrix metalloproteinase (MT1-MMP) is expressed on the endothelial tumor cells, including lung, gastric, colon and cervical carcinomas, gliomas and melanomas (Cerofolini et al., 2016).

**References**

Abbina, S., and Parambath, A. (2018). “PEGylation and its alternatives: A summary,” in Engineering of Biomaterials for Drug Delivery Systems (Elsevier), 363–376. doi:10.1016/B978-0-08-101750-0.00014-3

Abu Lila, A.S., Kiwada, H., and Ishida, T. (2013). The accelerated blood clearance (ABC) phenomenon: clinical challenge and approaches to manage. *J. Control. Release* 172, 38-47. doi:10.1016/j.jconrel.2013.07.026

Agrahari, V., and Agrahari, V. (2018). Facilitating the translation of nanomedicines to a clinical product: challenges and opportunities. *Drug Discov. Today* 23, 974–991. doi:10.1016/j.drudis.2018.01.047

Amoozgar, Z., and Yeo, Y. (2012). Recent advances in stealth coating of nanoparticle drug delivery systems. *Wiley Interdiscip. Rev. Nanomedicine Nanobiotechnology* 4, 219–233. doi:10.1002/wnan.1157

Assaraf, Y. G., Leamon, C. P., and Reddy, J. A. (2014). The folate receptor as a rational therapeutic target for personalized cancer treatment. Drug Resist. Updat. 17, 4–6. doi:10.1016/j.drup.2014.10.002

Bazak, R., Houri, M., El Achy, S., Kamel, S., and Refaat, T. (2015). Cancer active targeting by nanoparticles: a comprehensive review of literature. J. Cancer Res. Clin. Oncol. 141, 769-84. doi:10.1007/s00432-014-1767-3

Bertrand, N., Wu, J., Xu, X., Kamaly, N., and Farokhzad, O. C. (2014). Cancer nanotechnology: the impact of passive and active targeting in the era of modern cancer biology. *Adv. Drug Deliv. Rev*. 66, 2–25. doi: 10.1016/j.addr.2013.11.009

Cao, Z., and Jiang, S. (2012). Super-hydrophilic zwitterionic poly (carboxybetaine) and amphiphilic non-ionic poly (ethylene glycol) for stealth nanoparticles. *Nano Today* 7, 404–413. doi:10.1016/j.nantod.2012.08.001

Cao, Z., Yu, Q., Xue, H., Cheng, G., and Jiang, S. (2010). Nanoparticles for drug delivery prepared from amphiphilic PLGA zwitterionic block copolymers with sharp contrast in polarity between two blocks. *Angew. Chemie Int. Ed*. 49, 3771–3776. doi:10.1002/anie.200907079

Cassinelli, G., and Naggi, A. (2016). Old and new applications of non-anticoagulant heparin. *Int. J. Cardiol*. 212, S14-21. doi:10.1016/S0167-5273(16)12004-2

Cerqueira, B. B. S., Lasham, A., Shelling, A. N., and Al-Kassas, R. (2017). Development of biodegradable PLGA nanoparticles surface engineered with hyaluronic acid for targeted delivery of paclitaxel to triple negative breast cancer cells. *Mater. Sci. Eng. C* 76, 593–600. doi:1016/j.msec.2017.03.121

Cerofolini, L., Amar, S., Lauer, J. L., Martelli, T., Fragai, M., Luchinat, C., et al. (2016). Bilayer membrane modulation of membrane type 1 matrix metalloproteinase (MT1-MMP) structure and proteolytic activity. *Sci. Rep.* 6, 29511

Cosco, D., Mare, R., Paolino, D., Salvatici, M. C., Cilurzo, F., and Fresta, M. (2019). Sclareol-loaded hyaluronan-coated PLGA nanoparticles: Physico-chemical properties and in vitro anticancer features. *Int. J. Biol. Macromol*. 132, 550–557. doi:10.1016/j.ijbiomac.2019.03.241.

Cosco, D., Paolino, D., De Angelis, F., Cilurzo, F., Celia, C., Di Marzio, L., et al. (2015). Aqueous-core PEG-coated PLA nanocapsules for an efficient entrapment of water soluble anticancer drugs and a smart therapeutic response. *Eur. J. Pharm. Biopharm*. 89, 30–39. doi:10.1016/j.ejpb.2014.11.012

Cosco, D., Tsapis, N., Nascimento, T.L., Fresta, M., Chapron, D., Taverna, M., Arpicco, S., and Fattal, E. (2017). Polysaccharide-coated liposomes by post-insertion of a hyaluronan-lipid conjugate. *Colloids Surf. B Biointerfaces*. 158, 119-126. doi:10.1016/j.colsurfb.2017.06.029

Danhier, F., Feron, O., and Préat, V. (2010). To exploit the tumor microenvironment: Passive and active tumor targeting of nanocarriers for anti-cancer drug delivery. J. Control Release 148, 135-46. doi:10.1016/j.jconrel.2010.08.027

Dehaini, D., Fang, R. H., and Zhang, L. (2016). Biomimetic strategies for targeted nanoparticle delivery. *Bioeng. Transl. Med*. 1, 30–46. doi:10.1002/btm2.10004

Dobrovolskaia, M. A. (2015). Pre-clinical immunotoxicity studies of nanotechnology-formulated drugs: Challenges, considerations and strategy. *J. Control. Release* 220, 571–583. doi:10.1016/j.jconrel.2015.08.056

Dosio, F., Arpicco, S., Stella, B., and Fattal, E. (2016). Hyaluronic acid for anticancer drug and nucleic acid delivery. *Adv. Drug Deliv. Rev*. 97, 204–236. doi:10.1016/j.addr.2015.11.011

Engin, A. B., and Hayes, A. W. (2018). The impact of immunotoxicity in evaluation of the nanomaterials safety. *Toxicol. Res. Appl*. 2, 1-9. doi:10.1177/2397847318755579

Erkekoglu, P., and Baydar, T. (2014). Acrylamide neurotoxicity. *Nutr. Neurosci*. 17, 49–57. doi:10.1179/1476830513Y.0000000065

Fang, R. H., Kroll, A. V, and Zhang, L. (2015). Nanoparticle‐based manipulation of antigen‐presenting cells for cancer immunotherapy. *Small* 11, 5483–5496. doi:10.1002/smll.201501284

Farkona, S., Diamandis, E. P., and Blasutig, I. M. (2016). Cancer immunotherapy: the beginning of the end of cancer? *BMC Med*. 14, 73. doi:10.1186/s12916-016-0623-5

Folkman, J. (1971). Transplacental carcinogenesis by stilbestrol. *N Engl J Med.* 285, 404-405. doi: 10.1056/NEJM197108122850711

Gulati, N. M., Stewart, P. L., and Steinmetz, N. F. (2018). Bioinspired Shielding Strategies for Nanoparticle Drug Delivery Applications. *Mol. Pharm*. 15, 2900–2909. doi:10.1021/acs.molpharmaceut.8b00292

Hadjesfandiari, N., and Parambath, A. (2018). “Stealth coatings for nanoparticles: Polyethylene glycol alternatives,” in *Engineering of Biomaterials for Drug Delivery Systems*, 345–361. doi:10.1016/B978-0-08-101750-0.00013-1

Hannon, G., Lysaght, J., Liptrott, N. J., and Prina-Mello, A. (2019). Immunotoxicity Considerations for Next Generation Cancer Nanomedicines. *Adv. Sci*. 6, 1900133. doi:10.1002/advs.201900133.

Hoang Thi, T. T., Pilkington, E. H., Nguyen, D. H., Lee, J. S., Park, K. D., and Truong, N. P. (2020). The Importance of Poly(ethylene glycol) Alternatives for Overcoming PEG Immunogenicity in Drug Delivery and Bioconjugation. *Polymers (Basel).* 12. doi:10.3390/polym12020298.

Hu, C.-M. J., Zhang, L., Aryal, S., Cheung, C., Fang, R. H., and Zhang, L. (2011). Erythrocyte membrane-camouflaged polymeric nanoparticles as a biomimetic delivery platform. *Proc. Natl. Acad. Sci*. 108, 10980–10985. doi: 10.1073/pnas.1106634108

Hu, K., Zhou, H., Liu, Y., Liu, Z., Liu, J., Tang, J., et al. (2015). Hyaluronic acid functional amphipathic and redox-responsive polymer particles for the co-delivery of doxorubicin and cyclopamine to eradicate breast cancer cells and cancer stem cells. *Nanoscale* 7, 8607–8618. doi: 10.1039/C5NR01084E

Hu, Z.-D., Sun, Y., Guo, J., Huang, Y.-L., Qin, B.-D., Gao, Q., et al. (2014). Red blood cell distribution width and neutrophil/lymphocyte ratio are positively correlated with disease activity in primary Sjögren’s syndrome. *Clin. Biochem*. 47, 287–290. doi:10.1016/j.clinbiochem.2014.08.022

Huang, J., Zhang, H., Yu, Y., Chen, Y., Wang, D., Zhang, G., et al. (2014). Biodegradable self-assembled nanoparticles of poly (d, l-lactide-co-glycolide)/hyaluronic acid block copolymers for target delivery of docetaxel to breast cancer. *Biomaterials* 35, 550–566. doi:10.1016/j.biomaterials.2013.09.089

Humphries, J., Pizzi, D., Sonderegger, S. E., Fletcher, N. L., Houston, Z. H., Bell, C. A., et al. (2020). Hyperbranched Poly(2-oxazoline)s and Poly(ethylene glycol): A Structure-Activity Comparison of Biodistribution. *Biomacromolecules* doi:10.1021/acs.biomac.0c00765

Ilinskaya, A. N., and Dobrovolskaia, M. A. (2013). Nanoparticles and the blood coagulation system. Part II: safety concerns. *Nanomedicine (Lond).* 8, 969–981. doi:10.2217/nnm.13.49

Ishihara, T., Maeda, T., Sakamoto, H., Takasaki, N., Shigyo, M., Ishida, T., et al. (2010). Evasion of the accelerated blood clearance phenomenon by coating of nanoparticles with various hydrophilic polymers. *Biomacromolecules* 11, 2700–2706. doi:10.1021/bm100754e

Jaulin, N., Appel, M., Passirani, C., Barratt, G., and Labarre, D. (2000). Reduction of the uptake by a macrophagic cell line of nanoparticles bearing heparin or dextran covalently bound to poly(methyl methacrylate). *J. Drug Target*. 8, 165–172. doi:10.3109/10611860008996862

Jia, G., Cao, Z., Xue, H., Xu, Y., and Jiang, S. (2009). Novel zwitterionic-polymer-coated silica nanoparticles. *Langmuir* 25, 3196–3199. doi:10.1021/la803737c

Jiang, H., Wang, Q., and Sun, X. (2017). Lymph node targeting strategies to improve vaccination efficacy. *J. Control. Release* 267, 47–56. doi: 10.1016/j.jconrel.2017.08.009

Joh, D. Y., Zimmers, Z., Avlani, M., Heggestad, J. T., Aydin, H. B., Ganson, N., et al. (2019). Architectural Modification of Conformal PEG-Bottlebrush Coatings Minimizes Anti-PEG Antigenicity While Preserving Stealth Properties. *Adv. Healthc. Mater*. 8, e1801177. doi:10.1002/adhm.201801177

Jung, B., Shim, M.-K., Park, M.-J., Jang, E. H., Yoon, H. Y., Kim, K., et al. (2017). Hydrophobically modified polysaccharide-based on polysialic acid nanoparticles as carriers for anticancer drugs. *Int. J. Pharm*. 520, 111–118. doi:10.1016/j.ijpharm.2017.01.055

Khutoryanskiy, V. V (2018). Beyond PEGylation: Alternative surface-modification of nanoparticles with mucus-inert biomaterials. *Adv. Drug Deliv. Rev*. 124, 140–149. doi:10.1016/j.addr.2017.07.015

Knop, K., Hoogenboom, R., Fischer, D., and Schubert, U. S. (2010). Poly(ethylene glycol) in drug delivery: pros and cons as well as potential alternatives. *Angew. Chem. Int. Ed. Engl*. 49, 6288–6308. doi:10.1002/anie.200902672

Li, Y., and Boraschi, D. (2016). Endotoxin contamination: a key element in the interpretation of nanosafety studies. *Nanomedicine* 11, 269–287. doi: 10.2217/nnm.15.196

Li, M.H., Zong, H., Leroueil, P.R., Choi, S.K., and Baker, J.R., Jr. (2017). Ligand Characteristics Important to Avidity Interactions of Multivalent Nanoparticles. Bioconjug. Chem. 28, 1649-1657. doi:10.1021/acs.bioconjchem.7b00098

Liu, X., Xu, Y., Wu, Z., and Chen, H. (2013). Poly (N‐vinylpyrrolidone)‐modified surfaces for biomedical applications. *Macromol. Biosci*. 13, 147–154. doi:10.1002/mabi.201200269

Mohamed, M., Abu Lila, A. S., Shimizu, T., Alaaeldin, E., Hussein, A., Sarhan, H. A., et al. (2019). PEGylated liposomes: immunological responses. *Sci. Technol. Adv. Mater*. 20, 710–724. doi: 10.1080/14686996.2019.1627174

Muhammad, Q., Jang, Y., Kang, S. H., Moon, J., Kim, W. J., and Park, H. (2020). Modulation of immune responses with nanoparticles and reduction of their immunotoxicity. *Biomater. Sci*. 8, 1490–1501. doi:10.1039/C9BM01643K

Nascimento, T. L., Hillaireau, H., Noiray, M., Bourgaux, C., Arpicco, S., Pehau-Arnaudet, G., et al. (2015). Supramolecular organization and siRNA binding of hyaluronic acid-coated lipoplexes for targeted delivery to the CD44 receptor. *Langmuir* 31, 11186–11194. doi:10.1021/acs.langmuir.5b01979

Nieberler, M., Reuning, U., Reichart, F., Notni, J., Wester, H.-J., Schwaiger, M., et al. (2017). Exploring the Role of RGD-Recognizing Integrins in Cancer. *Cancers (Basel).* 9. doi:10.3390/cancers9090116

Obst, M., and Steinbüchel, A. (2004). Microbial degradation of poly(amino acid)s. *Biomacromolecules* 5, 1166–1176. doi:10.1021/bm049949u

Pandey, R. K., and Prajapati, V. K. (2018). Molecular and immunological toxic effects of nanoparticles. *Int. J. Biol. Macromol*. 107, 1278–1293. doi:10.1016/j.ijbiomac.2017.09.110

Pardoll, D. (2015). Cancer and the Immune System: Basic Concepts and Targets for Intervention. *Semin. Oncol*. 42, 523–538. doi:10.1053/j.seminoncol.2015.05.003

Park, H.-K., Lee, S. J., Oh, J.-S., Lee, S.-G., Jeong, Y.-I., and Lee, H. C. (2015). Smart nanoparticles based on hyaluronic acid for redox-responsive and CD44 receptor-mediated targeting of tumor. *Nanoscale Res. Lett*. 10, 288. doi:10.1186/s11671-015-0981-5

Park, J., and Babensee, J. E. (2012). Differential functional effects of biomaterials on dendritic cell maturation. *Acta Biomater*. 8, 3606–3617. doi:10.1016/j.actbio.2012.06.006

Park, K., Lee, G. Y., Kim, Y.-S., Yu, M., Park, R.-W., Kim, I.-S., et al. (2006). Heparin–deoxycholic acid chemical conjugate as an anticancer drug carrier and its antitumor activity. *J. Control. release* 114, 300–306. doi:10.1016/j.jconrel.2006.05.017

Park, W., Heo, Y.-J., and Han, D. K. (2018). New opportunities for nanoparticles in cancer immunotherapy. *Biomater. Res*. 22, 24. doi:10.1186/s40824-018-0133-y

Prabhu, V.V., and Devaraj, N. (2017). Epidermal Growth Factor Receptor Tyrosine Kinase: A Potential Target in Treatment of Non-Small-Cell Lung Carcinoma. J. Environ. Pathol. Toxicol. Oncol. 36, 151-158. doi: 10.1615/JEnvironPatholToxicolOncol.2017018341

Qi, Y., Simakova, A., Ganson, N. J., Li, X., Luginbuhl, K. M., Özer, I., et al. (2016). A brush-polymer conjugate of exendin-4 reduces blood glucose for up to five days and eliminates poly(ethylene glycol) antigenicity. *Nat. Biomed. Eng*. 1, 0002. doi:10.1038/s41551-016-0002

Ribatti, D. (2017). The concept of immune surveillance against tumors. The first theories. *Oncotarget* 8, 7175–7180. doi:10.18632/oncotarget.12739

Ryan, G. M., Kaminskas, L. M., and Porter, C. J. H. (2014). Nano-chemotherapeutics: Maximising lymphatic drug exposure to improve the treatment of lymph-metastatic cancers. *J. Control. Release* 193, 241–256. doi:10.1016/j.jconrel.2014.04.051

Schlesinger, M., and Bendas, G. (2015). Vascular cell adhesion molecule-1 (VCAM-1)-an increasing insight into its role in tumorigenicity and metastasis. *Int. J. cancer* 136, 2504–2514. doi:10.1002/ijc.28927

Shibuya, M. (2013). Vascular endothelial growth factor and its receptor system: physiological functions in angiogenesis and pathological roles in various diseases. *J. Biochem*. 153, 13–19. doi:10.1093/jb/mvs136

Socha, M., Lamprecht, A., El Ghazouani, F., Emond, E., Maincent, P., Barré, J., et al. (2008). Increase in the vascular residence time of propranolol-loaded nanoparticles coated with heparin. *J. Nanosci. Nanotechnol.* 8, 2369–2376. doi:10.1166/jnn.2008.081

Steichen, S.D., Caldorera-Moore, M., and Peppas, N.A. (2013). A review of current nanoparticle and targeting moieties for the delivery of cancer therapeutics. Eur. J. Pharm. Sci. 48, 416-27. doi:10.1016%2Fj.ejps.2012.12.006

Suk, J. S., Xu, Q., Kim, N., Hanes, J., and Ensign, L. M. (2016). PEGylation as a strategy for improving nanoparticle-based drug and gene delivery. *Adv. Drug Deliv. Rev*. 99, 28–51. doi:10.1016/j.addr.2015.09.012

Thomas, O. S., and Weber, W. (2019). Overcoming Physiological Barriers to Nanoparticle Delivery-Are We There Yet? *Front. Bioeng. Biotechnol*. 7, 415. doi:10.3389/fbioe.2019.00415

Tortorella, S., and Karagiannis, T. C. (2014). Transferrin receptor-mediated endocytosis: a useful target for cancer therapy. *J. Membr. Biol*. 247, 291–307. doi:10.1007/s00232-014-9637-0

Ulbrich, K., and Šubr, V. (2010). Structural and chemical aspects of HPMA copolymers as drug carriers. *Adv. Drug Deliv. Rev*. 62, 150–166. doi: 10.1016/j.addr.2009.10.007

Vangara, K. K., Liu, J. L., and Palakurthi, S. (2013). Hyaluronic acid-decorated PLGA-PEG nanoparticles for targeted delivery of SN-38 to ovarian cancer. *Anticancer Res*. 33, 2425–2434.

Ventola, C. L. (2017a). Cancer Immunotherapy, Part 1: Current Strategies and Agents. 42, 375–383. Available at: https://pubmed.ncbi.nlm.nih.gov/28579724.

Ventola, C. L. (2017b). Cancer Immunotherapy, Part 2: Efficacy, Safety, and Other Clinical Considerations. 42, 452–463. Available at: https://pubmed.ncbi.nlm.nih.gov/28674473.

Vetten, M. A., Yah, C. S., Singh, T., and Gulumian, M. (2014). Challenges facing sterilization and depyrogenation of nanoparticles: Effects on structural stability and biomedical applications. *Nanomedicine Nanotechnology, Biol. Med*. 10, 1391–1399. doi:10.1016/j.nano.2014.03.017

Viegas, T. X., Bentley, M. D., Harris, J. M., Fang, Z., Yoon, K., Dizman, B., et al. (2011). Polyoxazoline: chemistry, properties, and applications in drug delivery. *Bioconjug. Chem*. 22, 976–986. doi: 10.1021/bc200049d

Wilson, D. R., Zhang, N., Silvers, A. L., Forstner, M. B., and Bader, R. A. (2014). Synthesis and evaluation of cyclosporine A-loaded polysialic acid-polycaprolactone micelles for rheumatoid arthritis. *Eur. J. Pharm. Sci*. 51, 146–156. doi:10.1016/j.ejps.2013.09.013

Yadav, A. K., Mishra, P., Mishra, A. K., Mishra, P., Jain, S., and Agrawal, G. P. (2007). Development and characterization of hyaluronic acid-anchored PLGA nanoparticulate carriers of doxorubicin. *Nanomedicine* 3, 246–257. doi:10.1016/j.nano.2007.09.004

Yong, K.-T., Swihart, M. T., Ding, H., and Prasad, P. N. (2009). Preparation of gold nanoparticles and their applications in anisotropic nanoparticle synthesis and bioimaging. *Plasmonics* 4, 79–93. doi: https://doi.org/10.1007/s11468-009-9078-2

You, Y.-Z., and Oupický, D. (2007). Synthesis of temperature-responsive heterobifunctional block copolymers of poly(ethylene glycol) and poly(N-isopropylacrylamide). *Biomacromolecules* 8, 98–105. doi:10.1021/bm060635b

Youm, I., Agrahari, V., Murowchick, J. B., and Youan, B.-B. C. (2014). Uptake and cytotoxicity of docetaxel-loaded hyaluronic acid-grafted oily core nanocapsules in MDA-MB 231 cancer cells. *Pharm. Res*. 31, 2439–2452. doi: 10.1007/s11095-014-1339-x

Wang, B., Zhai, Y., Shi, J., Zhuang, L., Liu, W., Zhang, H., et al. (2017). Simultaneously overcome tumor vascular endothelium and extracellular matrix barriers via a non-destructive size-controlled nanomedicine. *J. Control. Release* 268, 225–236. doi:10.1016/j.jconrel.2017.10.029

Zamboni, F., Keays, M., Hayes, S., Albadarin, A. B., Walker, G. M., Kiely, P. A., et al. (2017). Enhanced cell viability in hyaluronic acid coated poly (lactic-co-glycolic acid) porous scaffolds within microfluidic channels. *Int. J. Pharm*. 532, 595–602. doi: 10.1016/j.ijpharm.2017.09.053

Zamboni, F., Vieira, S., Reis, R. L., Oliveira, J. M., and Collins, M. N. (2018). The potential of hyaluronic acid in immunoprotection and immunomodulation: chemistry, processing and function. *Prog. Mater. Sci*. 97, 97–122. doi:10.1016/j.pmatsci.2018.04.003

Zhang, G., Liao, Y., and Baker, I. (2010). Surface engineering of core/shell iron/iron oxide nanoparticles from microemulsions for hyperthermia. *Mater. Sci. Eng. C* 30, 92–97. doi: 10.1016/j.msec.2009.09.003

Zhang, P., Sun, F., Liu, S., and Jiang, S. (2016). Anti-PEG antibodies in the clinic: Current issues and beyond PEGylation. *J. Control. Release* 244, 184–193. doi: 10.1016/j.jconrel.2016.06.040

Zhuang, J., Fang, R. H., and Zhang, L. (2017). Preparation of particulate polymeric therapeutics for medical applications. *Small Methods* 1, 1700147. doi:10.1002/smtd.201700147

Zhuang, J., Holay, M., Park, J. H., Fang, R. H., Zhang, J., and Zhang, L. (2019). Nanoparticle Delivery of Immunostimulatory Agents for Cancer Immunotherapy. *Theranostics* 9, 7826–7848. doi:10.7150/thno.37216

| **Table S1. PEG alternatives** | | |
| --- | --- | --- |
| ***Polymer-based shielding approaches*** | | |
| *Compounds* | *Main properties* | *References* |
| *POEGMA* | Non-antigenicity and non-specific protein adsorption | Qi et al., 2016, Gulati et al., 2018, Joh et al., 2019. |
| *PAAm and PDMA* | Non-immunogenicity, low cost, stability over a wide range of pH intervals (pH 3-11) and ability to avoid protein absorption | You et al., 2007; Knop et al., 2010; Erkekoglu et al., 2014; Hadjesfandiari et al., 2018. |
| *PHPMA* | Low immunogenicity, low accumulation in liver and spleen | Abbina et al., 2018; Hoang Thi et al., 2018. |
| *PG* | Biocompatibility and low immunogenicity | Suk et al., 2016; Hadjesfandiari et al., 2018. |
| POX | Good solubility in hydrophilic and hydrophobic solvents; resistance to oxidative degradation | Viegas et al., 2011; Abbina et al., 2018; Khutoryanskiy et al., 2018. |
| PVP | Low degradation under UV or ultrasound irradiation; biocompatibility low immunogenicity. | Liu et al., 2013; Zhang et al., 2016. |
| ***Carbohydrates-based shielding approaches*** | | |
| *Polysialic acid* | Biocompatibility and biodegradability. | Wilson et al., 2014; Jung et al., 2017 Hoang Thi et al., 2018, Gulati et al., 2018. |
| *Hyaluronic acid* | Non toxicity, non immunogenicity, biocompatibility and biodegradability. | Nascimento et al., 2015; Zamboni et al., 2018; Cosco et al., 2017; Cosco et al., 2019. |
| *Heparin* | Biocompatibility and biodegradability. | Jaulin et al., 2000; Socha et al., 2008; Park et 2006; Cassinelli et al., 2016. |
| ***Other shielding approaches*** | | |
| *RBCs* | Low clearance and increased half-life of nanosystems. | Hu et al., 2011; Hu et al., 2013; Thomas et al., 2019. |
| *Poly (aminoacids)*  *PGA* | Approved by the FDA and EMA for food, pharmaceutical and cosmetic applications | Obst et al., 2004; Suk et al., 2016 |
| *Zwitterionic derivatives*  *(pCB) and (pSB)* | Low immunogenicity and high resistance to non-specific protein adsorption. | Jia et al., 2009; Yong et al., 2009; Zhang et al., 2010; Cao et al., 2010; Cao et al., 2012; Amoozar et al., 2013; Hoang Thi et al., 2018. |
